# Development and validation of clinical prediction models for personalized renal function monitoring in people with heart failure in primary care: the RENAL-HF study protocol

**DOI:** 10.1093/ehjdh/ztag055

**Published:** 2026-03-31

**Authors:** Alexandar Vincent-paulraj, Matthew J Carr, David A Jenkins, Bertram Muller-Myhsok, Mark Devonald, Jay Wright, Nefyn Williams, Niels Peek, Munir Pirmohamed, Darren M Ashcroft

**Affiliations:** Department of Pharmacology and Therapeutics, Institute of Systems Molecular and Integrative Biology, University of Liverpool, Liverpool, UK; Division of Pharmacy and Optometry, School of Health Sciences, Faculty of Biology, Centre for Pharmacoepidemiology and Drug Safety, Medicine and Health, University of Manchester, Manchester, UK; NIHR Greater Manchester Patient Safety Research Collaboration, University of Manchester, Manchester, UK; Division of Informatics, Imaging and Data Science, Faculty of Biology, Medicine and Health, University of Manchester, Manchester, UK; Department of Pharmacology and Therapeutics, Institute of Systems Molecular and Integrative Biology, University of Liverpool, Liverpool, UK; Liverpool University Hospitals NHS Foundation Trust, University of Liverpool, Liverpool, UK; Liverpool Heart and Chest Hospital NHS Foundation Trust, Liverpool, UK; Department of Primary Care and Mental Health, Institute of Population Health, University of Liverpool, Liverpool, UK; The Healthcare Improvement Studies Institute, Department of Public Health and Primary Care, University of Cambridge, Cambridge, UK; Department of Pharmacology and Therapeutics, Institute of Systems Molecular and Integrative Biology, University of Liverpool, Liverpool, UK; Liverpool University Hospitals NHS Foundation Trust, University of Liverpool, Liverpool, UK; Division of Pharmacy and Optometry, School of Health Sciences, Faculty of Biology, Centre for Pharmacoepidemiology and Drug Safety, Medicine and Health, University of Manchester, Manchester, UK; NIHR Greater Manchester Patient Safety Research Collaboration, University of Manchester, Manchester, UK

**Keywords:** Prediction model, Electronic health records, Heart failure, Renal function

## Abstract

**Aims:**

Heart failure (HF) is a growing problem in society with an ageing population and many patients with heart failure are affected by renal dysfunction. The RENAL-HF project aims to develop predictive risk models to support personalized renal function monitoring and treatment in patients with HF in primary care.

**Methods and results:**

This study will use electronic health records from the Clinical Practice Research Datalink (CPRD) database for patients who were diagnosed with HF. We will develop three prediction models—Mixed-effects model, Growth mixture model, and recurrent neural network-long short-term memory model to predict future worsening renal function, including events that lead to hospitalization, and death. Using an internal-external validation approach based on geographic region, we will choose the top-performing model using various metrics to evaluate the predictive performance.

**Conclusion:**

This protocol provides a detailed description of the methods used for developing and validating prognostic models for personalized renal function monitoring in people with HF in primary care.

**Protocol registration:**

The study and use of CPRD data were approved by the Independent Scientific Advisory Committee for Clinical Practice Research Datalink research (**Protocol Number: 22_001794).**

## Graphical Abstract

**Graphical Abstract:**
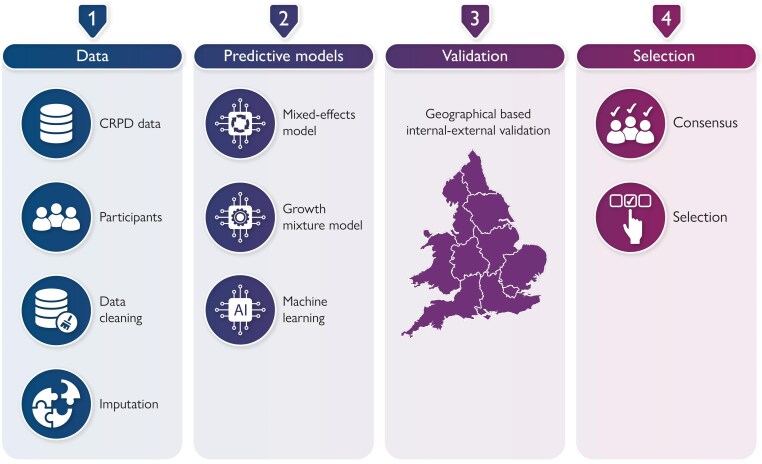

## Introduction

Approximately 15 million people in the Europe live with heart failure (HF), most of whom also have renal problems.^1^ In the UK as well as in other countries, most of these patients are managed in primary care. Modern therapy for people with HF comprises a combination of drugs, with dose titration being important to optimize control, and therefore improve prognosis. Sub-optimal treatment regimens and doses can lead to a progression of HF symptoms, and in turn worsen renal function. Conversely, over-treatment can also impair renal function. Either can lead to hospitalization and increases the risk of death.

There are no clear clinical guidelines on how renal function monitoring should be carried out in patients with HF. Even if population-based guidelines were available, they will be variably followed and may also be wasteful in terms of unnecessary testing for patients, increasing the burden on healthcare services and impairing the quality of life of patients. Hence, there is a need for tools that can inform primary care clinicians in the monitoring of renal function and guide treatment decisions.

The RENAL-HF project aims to develop an evidence-based system to support personalized renal function monitoring and treatment in patients with HF. Rooted in prediction technology, this system models the dynamic trajectory of renal function (serum creatinine) to predict the risk of worsening renal function (WRF) over a rolling 12-month horizon. Clinical prediction models (CPMs) are becoming increasingly important in modern clinical practice, informing healthcare professionals, patients, and families about the risks of outcomes, facilitating (shared) healthcare decision-making and improving health outcomes.^2^

There are several validated CPMs for the prediction of the onset and progression of chronic kidney disease (CKD) and for stratification of risk in patients with HF. These models focus on outcomes such as risk of admission and readmission, renal complications, and mortality.^3–10^ However, most of these models focus solely on static risk factors and do not directly deal with the dynamic changes in renal function over time. It is worth noting that comparative studies such as Christodoulou *et al*. showed the abilities of machine learning (ML) models and traditional statistical prediction methods in the clinical field.^11^ This study builds on this evidence base by explicitly comparing methods for the personalized monitoring of renal function in HF patients using statistical and ML frameworks.^12–16^

In this study, we provide a detailed scientific protocol for developing, validating, and comparing CPMs. This protocol directly compares three modelling strategies: mixed effects and growth mixture models (representing statistical approaches) and the recurrent neural network-long short-term memory (RNN-LSTM) model (an ML approach), applied to longitudinal electronic health records data from HF patients for the prediction of WRF over a 12-month horizon.

These models will be assessed using various evaluation metrics such as model performance, sustainability, interpretability, parsimony, novelty, implementation, and fairness. These metrics will help to identify the most suitable model to be used in a future clinical trial. This article describes the study protocol in three main stages: the development of WRF predictive models, the validation process of the models, and the selection of the final model for a clinical trial.

## Methods

**Figure 1:**
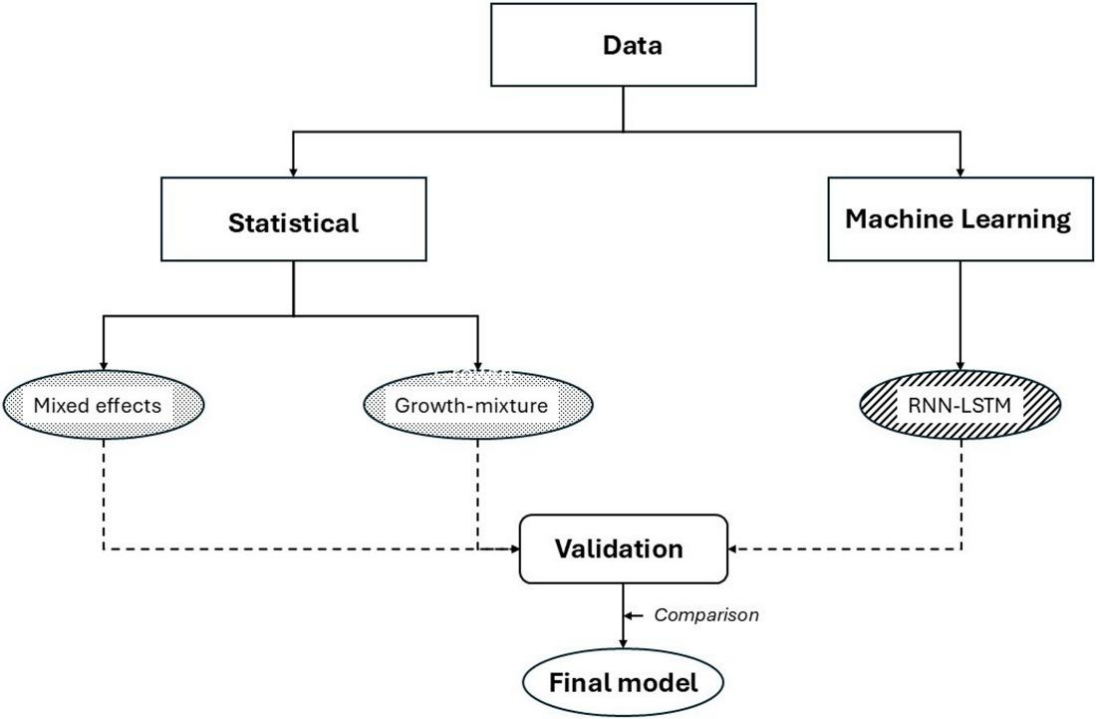
Flow chart describing overall view of study design.

The study is structured as follows: model development (conventional statistical and deep learning models), model validation (internal and external), and model comparison using consensus selection technique. The overview diagram (*Figure 1*) shows the data flow and the analysis steps, including which datasets are used in each step. This protocol complies with the relevant parts of the TRIPOD-AI statement on reporting of clinical predictive modelling studies using artificial intelligence.^17,18^ The completed TRIPOD-AI checklist is included in Supplementary File  *S1*.

### Data source

Using a retrospective cohort study design, we will use EHRs of HF patients to develop and validate predictive models of future renal function based on clinical and patient factors. The CPRD holds pseudonymized patient data from GP practices across the UK. In this study, we will be using records from the CPRD Aurum database for prediction model development.^19^ All predictor variables shall be derived from data collected routinely in the primary care setting. Outcome variables for hospitalization and mortality will be determined through the linkage to secondary care using hospital episode statistics and mortality data from the Office for National Statistics. In addition, the data will consist of socioeconomic status at the patient and practice-level (as a covariate and potentially for stratification) using the Index of Multiple Deprivation (IMD). In the CPRD databases, clinical diagnoses, medication, and laboratory test results are coded using a combination of Read v2, SNOMED CT, and local EMIS codes.

### Participants

The study population will consist of all patients with their first ever recorded diagnosis of HF between 1 January 2005 to 1 March 2022. We chose to use data after 2005 as the Quality and Outcomes Framework (QOF) was introduced in UK general practices in 2004 and resulted in improved recording in medical records of long-term conditions in primary care. All registered patients with a first Read v2 or SNOMED CT code for HF during the study period will be included in the study population. The clinical code lists will be made available in the GitHub repository: https://github.com/alxv/Renal-HF.

**Figure 2:**
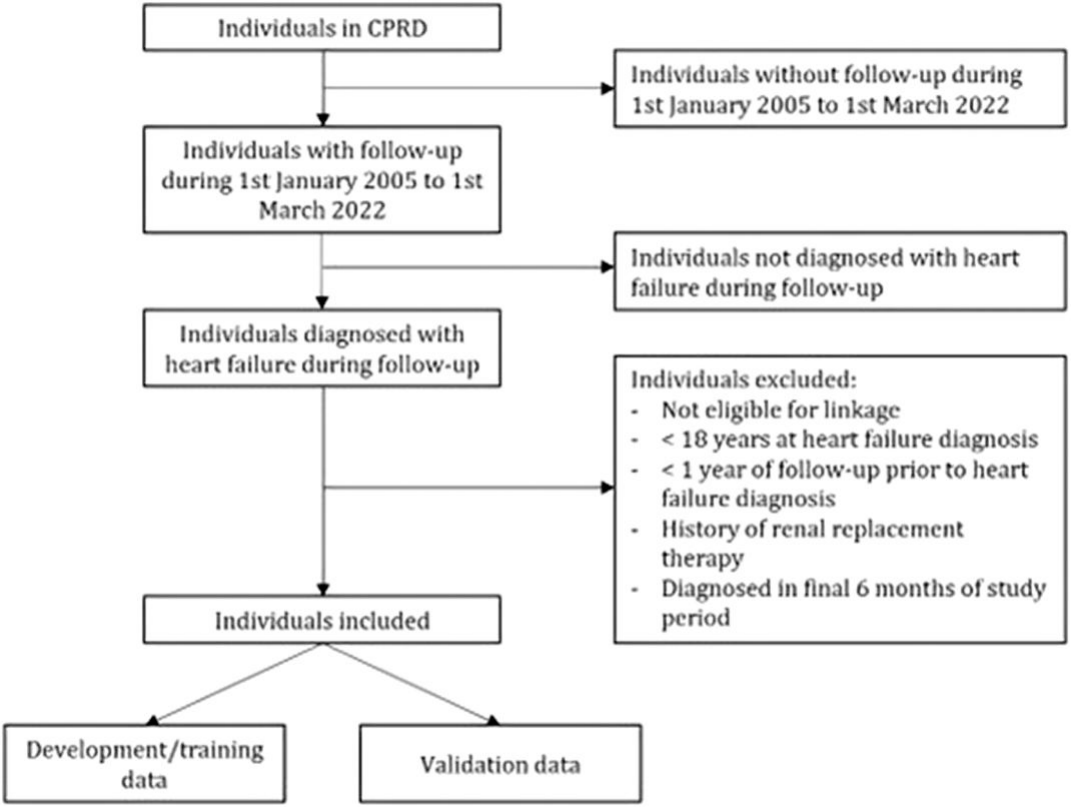
Flow diagram of patient inclusion/exclusion criteria.

There will be no restrictions to location in terms of the population sampled. A patient’s follow-up will commence on their index (onset of HF) date and terminate on either the last date of data collection (at the practice-level), the date that they transferred out of the practice, their date of death, or the end of the study, whichever happens first. Patients will be excluded from the study if they have had a history of renal replacement therapy (dialysis, filtration, or transplant) prior to their HF diagnosis. Patients who die or are lost to follow-up before the first valid serum creatinine (t_0_) is measured are excluded from longitudinal analysis as it is not possible to determine baseline renal function. A flow diagram of criteria for inclusion/exclusion is presented in *Figure 2*.

### Outcomes

The primary endpoint of the study will be worsening of renal function. Early detection or prediction of WRF provides an opportunity for intervention to prevent progression to renal failure and acute kidney injury (AKI) and potentially prevent hospitalization and death. Serum creatinine was selected as the primary biomarker for modelling. While a retrospective glomerular filtration rate (eGFR) recalculation using a single formula (CKD-EPI 2021) was a way of standardizing the historical data, we decided on raw serum creatinine. This approach avoids any bias. The raw serum creatinine allows an ML model to learn the effect of age and gender on renal trajectories directly from raw data rather than being constrained by fixed eGFR coefficients. In addition, a 12-month prediction period has been chosen to align with routine chronic disease monitoring intervals in primary care, thereby bridging the gap between acute injury (AKI) and long-term disease progression. Therefore, WRF is defined by primary care data if any of the following occur within 12 months: (i) 30% increase in serum creatinine or (ii) 26.5 µmol/L increase in serum creatinine.

Secondary outcomes will include hospitalization for AKI, major adverse cardiovascular events (MACE) and all-cause mortality. These outcomes are included specifically to consider competing risks and to recognize that patients with HF may experience cardiovascular events or death before renal monitoring.

### Predictors

Data shall be presented in longitudinal panel data format. Although cohort eligibility is determined at the date of diagnosis of HF (index date), the longitudinal model trajectory is based on the first available serum creatinine (*t*_0_) measurement in each patient. If there is a gap between the index date and the first creatinine measurement, the static baseline covariates are shifted to *t*_0_. The longitudinal data will contain demographic information, lifestyle factors, co-morbidities and medication use associated with HF, renal decline, and mortality following a review of the literature on HF and renal function monitoring.^20–26^ The major factors for code list preparation are provided in *Table 1*. A full list of definitions for the planned predictor variables is provided (along with definitions of units of measurement, categories, and acceptable value ranges) in Supplementary File  *S2*. The code lists used to extract the relevant data and define the candidate predictors will be developed, curated, and validated by clinical experts within the RENAL-HF project team. All code lists will be made available in the GitHub repository: https://github.com/alxv/Renal-HF. The models will use two categories of predictors. Static predictors are baseline demographics (age, sex, ethnicity, deprivation index) and comorbidities observed at the index date. Dynamic predictors will be time-varying variables updated at each measurement point, including serum creatinine, other longitudinal laboratory tests (potassium, sodium, urea) and the status of active medicinal products. All models utilize data available up to the moment of prediction (*t*) to forecast outcomes at *t* + 1. Future data are never used for model development at time t.

**Table 1:** Major factors for code list preparation

Description	Factors
**Cardiovascular**	Blood pressure, hypertension, smoking status, atrial fibrillation, and ischaemic heart disease
**Renal**	Chronic kidney disease status, history of AKI, renovascular disease, dialysis, nephrectomy, proteinuria, nephritis, and anaemia
**Comorbidities**	Chronic liver disease, diabetes, systemic lupus erythematosus, and peripheral vascular disease
**Lab tests**	Haemoglobin, potassium, albumin, sodium, mean corpuscular volume, and urea
**Medication**	Non-steroidal anti-inflammatory drugs, drugs acting on the angiotensin system, specific antimicrobials, and immunosuppressants

### Sample size

The sample size calculation for the statistical models was done in accordance with Riley *et al*. to ensure adequate performance and avoid overfitting based on the expected number of predictors and prevalence of outcomes.^27^ The calculation was based on an expected prevalence of outcome of ∼25%, approximately 100 candidate predictor parameters (including non-linearity) and a target reduction factor of 0.9. For the RNN-LSTM ML approach, at least 36 000 patients are required based on the number of network parameters, published precedents in similar studies, and practical feasibility. The sample size for ML models is still under active research.^28^

### Missing data

As the longitudinal data will be anchored on the available serum creatinine measurements for each patient, we will not have any missing outcome data. Missing data for the candidate predictors will be addressed using a non-parametric imputation approach using the last measured value for a given patient as a covariate (when available) alongside other concurrent measurements.^29^ The imputation will be undertaken using the R package missForest which utilizes an iterative random forest process.

### Descriptive analyses

Descriptive data analyses will be carried out on the study cohort.^30,31^ We will describe the baseline demographics and characteristics of the patients with HF including the distributions of age, gender, ethnicity and IMD, and the prevalence of comorbidities and medication use at baseline, i.e. at HF diagnosis. We will also describe the temporal prevalence of HF across the GP practices involved in the study and assess the baseline distributions of serum creatinine and eGFR within various strata.

### Prediction models

To predict serum creatinine trends, we will perform and compare three different modelling strategies. Modelling a continuous trajectory allows us to capture the rate of decline and dynamically recalculate the risk of WRF in relation to the change in baseline patient. This approach offers greater clinical utility than binary classification alone.

1. **Mixed-effects model**: The longitudinal component is based on a linear mixed-effects regression model. Variable selection is done with the least-absorption and selection operator (LASSO) to avoid overfitting by penalizing regression coefficients.
2. **Growth mixture model:** GMM is used to identify latent subgroups (classes) of a population that follow different growth trajectories. To mitigate overfitting in the GMM, the optimal number of latent classes (K) will be determined using the Bayesian information criterion and Akaike information criterion, with a minimum class size constraint enforced to ensure generalizability.
3. **RNN-LSTM:** Deep learning model using LSTM will be developed to handle complex nonlinear time dependencies. The model architecture consists of two LSTM layers (64 units each) with a dense output layer. To address the specific data requirements of neural nets, continuous variables (e.g. creatinine, urea) will be log transformed or standardized to ensure effective optimization, while clinical scales will be used in statistical models. To meet the specific data requirements of ML models, the continuous variable log transformation or standardization will be performed. Overfitting in RNN-LSTM is mitigated by early stopping, where training is stopped when the validation loss does not improve after a certain number of epochs (patience), in combination with drop-out regularization layers.

### Statistical modelling

The longitudinal component will consist of a linear mixed-effects regression model for the repeated measures of serum creatinine (at the patient level). To select variables for inclusion in the longitudinal sub model, we will use the LASSO proposed by Tibshirani (1996) which is based on penalized regression and uses an *L*_1_-penalty on the regression coefficients.^32^ Selection will be undertaken using a Gaussian link function in the R package glmmLasso which combines gradient ascent optimization with the Fisher scoring algorithm^33^. The mixed effects model (incorporating those variables selected via the LASSO) will be estimated using the R package nlme—as this provides the requisite outputs to be applied as arguments in the subsequent joint modelling phase. A GMM is a type of finite mixture model that assumes different subgroups (classes) in the population follow different growth trajectories. It combines features of growth curve modelling and mixture modelling. For a GMM, we assume there are *K* latent classes in the population, and each class *k* has its own growth model with distinct parameters. Within each class *k*, the model for the serum creatinine response variable is as follows:


$$y_{ij}=x_{ij}\beta_{k}+z_{ij}u_{ki}+\epsilon_{kij}$$

where

-
$x_{ij}$ and $z_{ij}$ are as defined previously.
-
$\beta_{k}$ is the column vector of fixed-effects coefficients for class *k*.
-
$u_{ki}$ is the column vector of random effects for the *i*th patient in class *k*.
-
$\epsilon_{kij}∼N(0,\sigma_{k}^{2})$ is the residual error for the *i*th patient at the *j*th measurement point in class *k*.

We will employ a joint modelling framework to link the longitudinal serum creatinine trajectory to the time to event outcome. The longitudinal sub-models (mixed-effects model or GMM) will characterize the renal trajectory. The survival sub-model will account for the informative censoring caused by competing risks such as death and MACE.

### Outcome predictions

**Figure 3:**
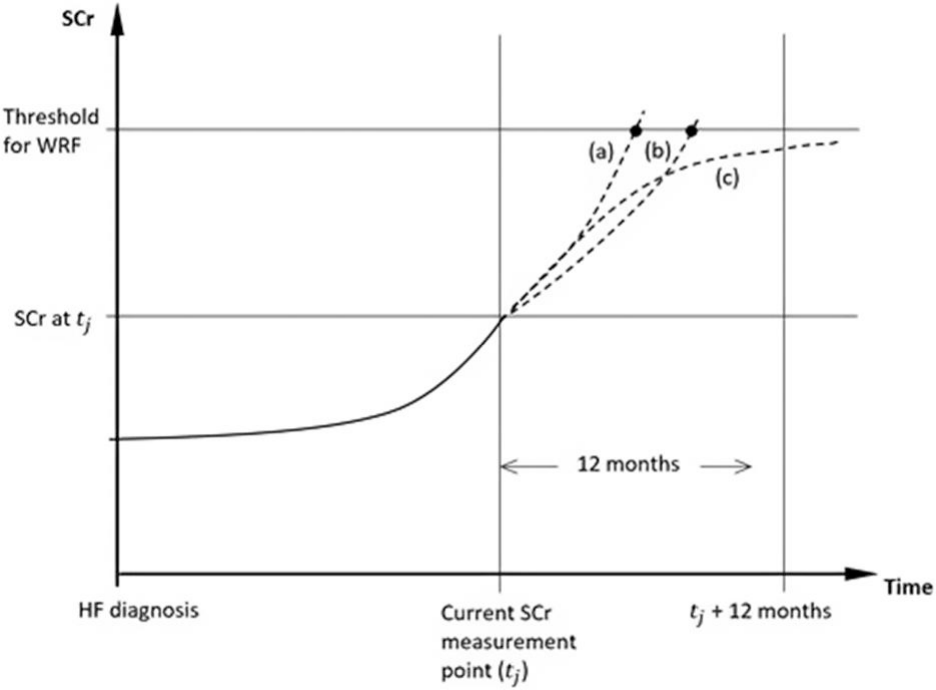
An illustration of serum creatinine pathway prediction using statistical models.

**Figure 4:**
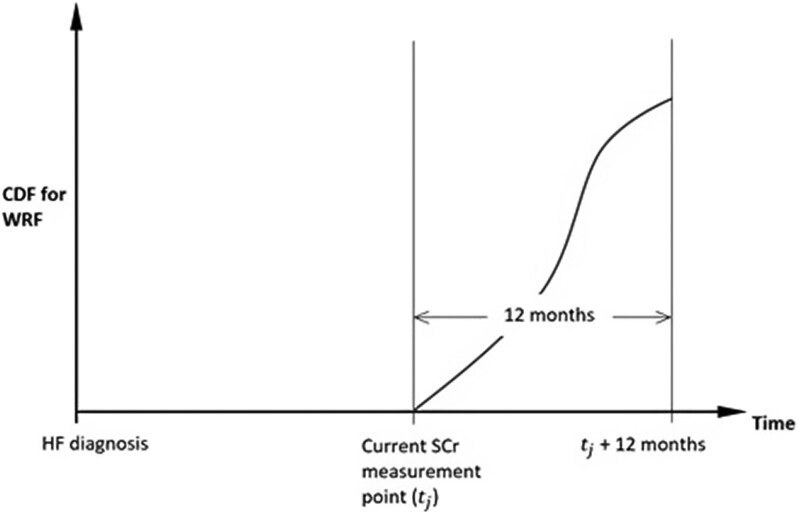
Probability distribution for WRF within 12 months from the current serum creatinine measurement point.

We have created an approach, which is described in Supplementary File  *S3*, to forecast a patient’s future serum creatinine trajectory. *Figure 3* shows the application of the statistical models in predicting future serum creatinine measurements. Predicted pathways (a) and (b) cross the WRF threshold within 12 months, while pathway (c) does not. By forecasting potential creatinine trajectories, we could construct an empirical cumulative distribution for the probability of WRF over time (*Figure 4*). Using the estimated joint model, we will make dynamic predictions for competing secondary outcomes by deriving cumulative event probabilities based on a patient’s observed serum creatinine process, covariate history, and outcome-free survival up to the current time. This will be achieved via Markov chain simulation (Metropolis-Hastings algorithm) and a Monte Carlo process.^34^

Secondary outcome risks will be assessed using (i) cause-specific Cox regression models with ridge penalization and (ii) Cox proportional hazard models to subsets of patients who remain in the risk set—i.e. those who have not experienced secondary outcomes or been censored before pre-specified landmark time points.^35^ Additionally, we will explore joint latent class model, where patients are assumed to belong to a single latent class, defining the association between the longitudinal and survival sub models.^36^

### Machine learning modelling

#### Data pre-processing for machine learning

The same longitudinal data from electronic health records that will be used to develop the statistical models will be used to develop the RNN-LSTM model. Although the first stages of data preparation are the same for both modelling strategies, more preprocessing is needed for the ML approach.^37,38^ For neural networks to process data, all variables need to be transformed into a numerical format. Using one-hot encoding, categorical variables like smoking status and gender will be converted to binary indicator variables (0,1) for every category level. We will use log transformations to normalize the distributions of continuous variables, such as serum creatinine, urea, potassium, and sodium, lessen the effect of outliers, and enhance model convergence during training. We will use masking and padding strategies to produce consistent sequence lengths. This will help to solve the problem of inconsistent time series lengths across patient records.^39^ Specifically, short sequences will have zeros padded at specific locations, and the neural network’s masking layer will make sure that these padding values don’t affect the model’s predictions. The details of RNN-LSTM model development are provided in Supplementary File  *S4*.

### Dynamic renal test recommendation system

Dynamic renal test recommendation system module aims to dynamically recommend renal tests based on inputs from the main model. The system proposes time to renal blood test using NICE (National Institute for Health and Care Excellence) guideline as backbone. The proof-of-concept R code is provided in Supplementary File  *S4*. The RNN-LSTM model (Main model) would provide estimations of future serum creatine levels based on historic data of a patient. The NICE guideline offers thresholds and requirement for assessing renal function and identifying WRF. We will adopt the RNN-LSTM prediction model's output to inform the NICE guideline recommendations.

### Explainability

We will use SHAP (SHapley Additive exPlanations) values to apply explainability techniques to address the “black box” nature of deep learning models^40^. To handle sequential data, we will modify the SHAP framework for the time-series LSTM model. This will involve calculating feature importance at each time step and then combining these values to determine both temporal patterns of influence and global feature importance. This approach will quantify how each input variable contributes to predictions across different time points. With this method, the contribution of each input variable to predictions at various time points will be quantified.

### Model assessment

Model assessment refers to the evaluation of predictive accuracy, discrimination, and calibration in a development dataset. On the other hand, validation evaluates the performance of the model in completely invisible (non-local or regionally external) datasets. For each of the three modelling strategies, both assessment and validation steps will be carried out. Detailed mathematical definitions of these metrics are provided in Section 3 of Supplementary File  *S3*. The assessment of these parameters will be as follows:

#### Predictive accuracy

Assessed using the root mean squared prediction error (RMSPE) to quantify the average distance between predicted and observed creatinine levels.

#### Calibration

Evaluating the agreement between predicted probabilities and observed event rates across risk groups.

#### Discrimination

Using the C-statistic to measure the model’s ability to distinguish between patients who will and will not experience WRF.

#### Clinical utility

To assess the practical value of the models, we will perform decision curve analysis. This method calculates the ‘Net Benefit’ of using the model to guide monitoring decisions across a range of threshold probabilities, comparing it against default strategies (e.g. monitoring everyone or no one). We will use methods described in previous research studies to calculate net benefits.^41,42^

### Validation

**Figure 5:**
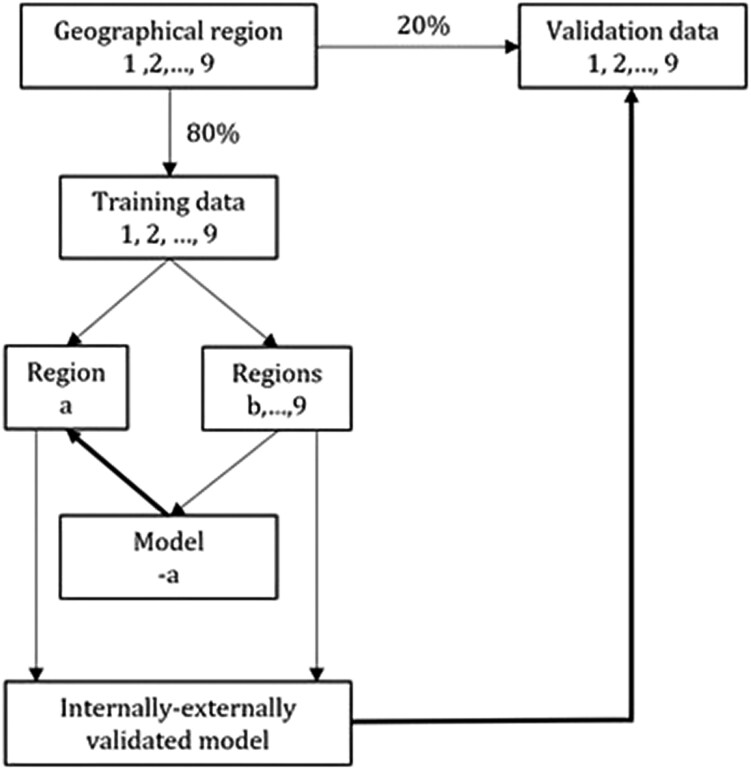
Proposed regional internal-external cross-validation and subsequent external validation.

For validation purposes, we will use a combination of split-sample and internal-external validation approaches.^43^ Internal-external validation will be used to demonstrate external validity.^44^ To prevent data leakage, the entire modelling pipeline including imputation (Random Forest), variable transformation, and feature selection will be performed independently within each training fold before being applied to the validation region. The CPRD Aurum data are drawn from nine geographical regions within England. By region, the data will be split, at the practice level, on a 4:1 ratio for development and external validation purposes. The internal validation will be carried out by considering eight out of nine regions for training and the left-out region will be used for validation. This process will result in nine models that are internally validated. *Figure 5* illustrates one such internal-external validation where region a is left out to cross validate a model developed in the other regions. The final model would be trained on all development data (nine regions) and will be validated against the holdout validation data (20%).

The performance measures will be pooled using random effect meta-analyses.^45^ This will also facilitate assessments of between-region variability (heterogeneity) of performance, which will be quantified by the between-region standard deviation of each model performance measure.^46^ Following meta-analysis, the performance measures will be reported as point estimates with 95% confidence and prediction intervals. The confidence intervals will quantify the precision of the average performance of the models across the different geographical regions. The prediction intervals will account for the heterogeneity across regions and will therefore function as an indicator of the expected performance within a specific region and illustrate the various performance measures that will be derived and meta-analysed for the primary outcome.

### Model selection

The final model selection process will include an assessment of transparency and explainability of the three candidate models. A consensus exercise will be undertaken using the RAND/UCLA appropriateness method.^47^ In addition, our model selection criteria will be accuracy, generalizability, implementability, interpretability, novelty and innovation, simplicity, and sustainability. The model selection criteria are detailed in Supplementary File  *S5*. Accuracy is quantified using RMSPE on both training and independent datasets, while fairness ensures that predictions are equitably accurate across diverse demographic groups. In this study protocol, we will be using the predictive parity (or calibration fairness) framework. Generalizability assesses the model's ability to function far beyond its development context, and implementability assesses how easy it is to integrate into the practice for decision-making. Interpretability refers to the overall clarity of the model for effective correction and bias screening, without focusing on individual case explanations. Novelty and innovation assess the introduction of new techniques or methods, while parsimony favours simpler models with fewer variables to reduce the risk of overfitting. Finally, sustainability ensures that the model can be updated effectively and maintained with minimal expenditure on resources in the event of new data becoming available.

## Discussion

This protocol outlines our planned study for creating and validating a prediction model for future serum creatinine trends in people with HF. Conventional renal monitoring strategies utilizing static serum creatinine or estimated GFR values often fail to capture the dynamic nature of renal function in HF patients. Our framework provides a clear guideline for developing and validating both statistical and ML algorithms for clinical implementation.

Our protocol addresses several challenges with respect to predictive model development. Serum creatinine is influenced by factors such as muscle mass and hydration. Although our model development process will focus on a specific cohort (UK), external validation in diverse populations will be required to confirm generalizability. Looking ahead, prospective studies will be needed to determine whether model-based interventions can reduce adverse effects on the kidney, optimize HF therapy titration, and improve overall clinical outcomes. This will be tested in a planned randomized controlled trial in the future.

Our protocol sets out a clear, step-by-step methodology for developing and validating a predictive model for monitoring renal function in HF patients. The protocol describes standardized data collection, obvious inclusion/exclusion criteria, rigorous preprocessing (including dealing with missing data), and the development of both statistical and ML algorithms. We also outline plans for internal and external validation across diverse populations and describe how the final model could be integrated with electronic health platforms to enable real-time decision support. This comprehensive methodological framework not only aims to develop an effective forecasting tool but also sets a reproducible standard for high-quality research in renal function monitoring in HF.

### Limitations

This study has few limitations, particularly its reliance on EHR data from the UK, which may affect its generalizability to other health systems and ethnic groups. Furthermore, while we use the most up-to-date internal and external validation, in future work, true external validation in non-UK environments will be required. Other limitations include the potential for misclassification or underestimation of lifestyle and socio-demographic covariates. If the distribution of data shifts over time or after application to new clinical settings, a dynamic update or recalibration strategy would be required.

Finally, we’re using non-parametric random forest imputation to fill in the missing data. This is because of computational constraints in training deep learning models, where single imputation is preferred to multiple imputation. We recognize that this approach may underestimate the uncertainty (standard errors) associated with the expected values compared to a multi-method approach.

### RENAL-HF consortium

Benjamin Brown, Carolyn Lees, Girvan Burnside, Jennifer Downing, Tim Williams, Lynn Hedgecoe, Bridget Young, Dyfrig Hughes, and Christopher Arden Armitage.

## Supplementary Material

ztag055_Supplementary_Data

## Data Availability

Data can be obtained from third parties and is not publicly available. Electronic health records are considered ‘sensitive’ data in the UK under Data Protection Act and cannot be shared through public statement due to restrictions in place to protect patient confidentiality as part of information management.
