# Supplementary material for: Development and validation of clinical prediction models for personalized renal function monitoring in people with heart failure in primary care: the RENAL-HF study protocol
Source: Eur Heart J Digit Health. 2026 Mar 31;7(4):ztag055. doi: 10.1093/ehjdh/ztag055 (PMC13131987; doi:10.1093/ehjdh/ztag055)
Supplement: ztag055_Supplementary_Data [file ztag055_supplementary_data.zip › S2.pdf]

Supplementary File 2: Study variables

Group	Variable	Description	Units	Range/categories
Fixed demographics/ characteristics and registration details	index	[Administrative: for model fitting]	integer	≥ 1
	myid	[Administrative: for model fitting]	integer	≥ 1
	patid	Patient ID (CPRD)		
	pracid	General practice ID		
	region	Region in which the practice is located	categorical	1 - 9
	imd10_prac	Practice-level Index of Multiple Deprivation (IMD) decile	decile	1 - 10
	indexdate	Date of HF diagnosis	date	DD-MM-YYYY
	gender	Gender	categorical	1 (Male); 2 (Female)
	ethnicity_hes	Ethnicity (from secondary care records)	categorical	White/Asian/Black/Mixed/Other/Unknown
	ethnicity_cprd	Ethnicity (from primary care records)	categorical	White/Asian/Black/Mixed/Other/Unknown
	ethnicity	Ethnicity	categorical	White/Asian/Black/Mixed/Other/Unknown
	yob	Year of birth	year	YYYY
	imd10_pat	Patient-level Index of Multiple Deprivation (IMD) decile	decile	1 - 10
	regstartdate	Registration start date with general practice	date	DD-MM-YYYY
	regenddate	Registration end date with general practice	date	DD-MM-YYYY
	ons_ddate	Date of death	date	DD-MM-YYYY
	lcd	Last date that data was collected from the general practice	date	DD-MM-YYYY
Renal replacement therapy (RRT) - Exclusion criteria and censoring variables	dialysis_dfirst	Date of first dialysis (in primary or secondary care records)	date	DD-MM-YYYY
	filtration_dfirst	Date of first renal filtration (in primary or secondary care records)	date	DD-MM-YYYY
	transplant_dfirst	Date of first renal transplant (in primary or secondary care records)	date	DD-MM-YYYY
	rrt_dfirst	Date of first recorded RRT	date	DD-MM-YYYY
	rrt_prior	RRT prior to HF diagnosis	binary	0,1
Serum creatinine measurements (panel data arrangement)	insample	Indicator if observation eligible for inclusion in analyses	binary	0,1
	obsindex	Patient's (in sample) observation index	integer	≥ 1
	obsdate	Serum creatinine observation date	date	DD-MM-YYYY
	serum_creatinine	Serum creatinine measurement	µmol/L	18 to 3000
Time and age variables	_time	Time since first (insample) creatinine measurement, i.e., on/after HF diagnosis	years	≥ 0
	_time_since_HF	Time since HF diagnosis	years	≥ 0
	_time_calendar	Calendar time (origin: 1st January 2005)	years	≥ 0
	_age	Age (approximate) at serum creatinine observation date (obsdate)	years	≥ 18
	_age_at_HF	Age (approximate) at HF diagnosis	years	≥ 18
Time-varying demographics/ characteristics	bmi_prior	Most recent Body Mass Index (BMI) measurement on/before HF diagnosis	categorical	Underweight/Normal/Overweight/Obese/Unknown
	bmi_prior_lag	Lag between 'bmi_prior' and HF diagnosis	days	≥ 0 OR Missing
	bmi_cat	Most recent Body Mass Index (BMI) measurement on/before current observation date	categorical	Underweight/Normal/Overweight/Obese/Unknown
	bmi_cat_lag	Lag between 'bmi_cat' and current observation date	days	≥ 0 OR Missing
	smoking_prior_cat	Most recent Smoking Status on/before HF diagnosis	categorical	Never/Ex/Current/Unknown
	smoking_prior_cat_lag	Lag between 'smoking_prior_cat' and HF diagnosis	days	≥ 0 OR Missing
	smoking_cat	Most recent Smoking Status on/before current observation date	categorical	Never/Ex/Current/Unknown
	smoking_cat_lag	Lag between 'smoking_cat' and current observation date	days	≥ 0 OR Missing
eGFR	e_gfr	Estimated glomerular filtration rate (eGFR)	ml/min/1.73m^2	> 0
Acute kidney injury (AKI)	aki_prior	Any AKI prior to HF diagnosis	binary	0,1
	aki_hx	Any AKI on/before current observation date	binary	0,1
	aki_hx7	Any AKI at least 7 days before current observation date	binary	0,1
	aki_hx30	Any AKI at least 30 days before current observation date	binary	0,1
Clinical measures	me_bnp_prior	Most recent Brain Natriuretic Peptide measurement on/before HF diagnosis	pg/ml	1 - 10,000
	me_bnp_prior_lag	Lag between 'me_bnp_prior' and HF diagnosis	days	≥ 0 OR Missing
	me_bnp	Most recent Brain Natriuretic Peptide measurement on/before current observation date	pg/ml	1 - 10,000
	me_bnp_lag	Lag between 'me_bnp' and current observation date	days	≥ 0 OR Missing
	me_dbp_prior	Most recent Diastolic Blood Pressure measurement on/before HF diagnosis	mm/Hg	20 - 200
	me_dbp_prior_lag	Lag between 'me_dbp_prior' and HF diagnosis	days	≥ 0 OR Missing
	me_dbp	Most recent Diastolic Blood Pressure measurement on/before current observation date	mm/Hg	20 - 200
	me_dbp_lag	Lag between 'me_dbp' and current observation date	days	≥ 0 OR Missing
	me_sbp_prior	Most recent Systolic Blood Pressure measurement on/before HF diagnosis	mm/Hg	30 - 300

	me_sbp_prior_lag	Lag between 'me_sbp_prior' and HF diagnosis	days	≥ 0 OR Missing
	me_sbp	Most recent Systolic Blood Pressure measurement on/before current observation date	mm/Hg	30 - 300
	me_sbp_lag	Lag between 'me_sbp' and current observation date	days	≥ 0 OR Missing
	me_haemogl_prior	Most recent Haemoglobin measurement on/before HF diagnosis	g/L	10 - 260
	me_haemogl_prior_lag	Lag between 'me_haemogl_prior' and HF diagnosis	days	≥ 0 OR Missing
	me_haemogl	Most recent Haemoglobin measurement on/before current observation date	g/L	10 - 260
	me_haemogl_lag	Lag between 'me_haemogl' and current observation date	days	≥ 0 OR Missing
	me_heartrate_prior	Most recent Heart Rate measurement on/before HF diagnosis	bpm	20 - 250
	me_heartrate_prior_lag	Lag between 'me_heartrate_prior' and HF diagnosis	days	≥ 0 OR Missing
	me_heartrate	Most recent Heart Rate measurement on/before current observation date	bpm	20 - 250
	me_heartrate_lag	Lag between 'me_heartrate' and current observation date	days	≥ 0 OR Missing
	me_mcv_prior	Most recent Mean Corpuscular Volume (MCV) measurement on/before HF diagnosis	fL	50 - 150
	me_mcv_prior_lag	Lag between 'me_mcv_prior' and HF diagnosis	days	≥ 0 OR Missing
	me_mcv	Most recent Mean Corpuscular Volume (MCV) measurement on/before current observation date	fL	50 - 150
	me_mcv_lag	Lag between 'me_mcv' and current observation date	days	≥ 0 OR Missing
	me_nt_probnp_prior	Most recent B-type Natriuretic Peptide (NT-pro BNP) measurement on/before HF diagnosis	pg/mL	1 - 30,000
	me_nt_probnp_prior_lag	Lag between 'me_nt_probnp_prior' and HF diagnosis	days	≥ 0 OR Missing
	me_nt_probnp	Most recent B-type Natriuretic Peptide (NT-pro BNP) measurement on/before current observation date	pg/mL	1 - 30,000
	me_nt_probnp_lag	Lag between 'me_nt_probnp' and current observation date	days	≥ 0 OR Missing
	me_albumin_prior	Most recent Serum Albumin measurement on/before HF diagnosis	g/L	1 - 60
	me_albumin_prior_lag	Lag between 'me_albumin_prior' and HF diagnosis	days	≥ 0 OR Missing
	me_albumin	Most recent Serum Albumin measurement on/before current observation date	g/L	1 - 60
	me_albumin_lag	Lag between 'me_albumin' and current observation date	days	≥ 0 OR Missing
	me_potassium_prior	Most recent Serum Potassium measurement on/before HF diagnosis	mmol/L	1 - 10
	me_potassium_prior_lag	Lag between 'me_potassium_prior' and HF diagnosis	days	≥ 0 OR Missing
	me_potassium	Most recent Serum Potassium measurement on/before current observation date	mmol/L	1 - 10
	me_potassium_lag	Lag between 'me_potassium' and current observation date	days	≥ 0 OR Missing
	me_sodium_prior	Most recent Serum Sodium measurement on/before HF diagnosis	mmol/L	80 - 200
	me_sodium_prior_lag	Lag between 'me_sodium_prior' and HF diagnosis	days	≥ 0 OR Missing
	me_sodium	Most recent Serum Sodium measurement on/before current observation date	mmol/L	80 - 200
	me_sodium_lag	Lag between 'me_sodium' and current observation date	days	≥ 0 OR Missing
	me_urea_prior	Most recent Serum Urea measurement on/before HF diagnosis	mmol/L	1 - 120
	me_urea_prior_lag	Lag between 'me_urea_prior' and HF diagnosis	days	≥ 0 OR Missing
	me_urea	Most recent Serum Urea measurement on/before current observation date	mmol/L	1 - 120
	me_urea_lag	Lag between 'me_urea' and current observation date	days	≥ 0 OR Missing
	me_uricacid_prior	Most recent Uric Acid measurement on/before HF diagnosis	µmol/L	1 - 1000
	me_uricacid_prior_lag	Lag between 'me_uricacid_prior' and HF diagnosis	days	≥ 0 OR Missing
	me_uricacid	Most recent Uric Acid measurement on/before current observation date	µmol/L	1 - 1000
	me_uricacid_lag	Lag between 'me_uricacid' and current observation date	days	≥ 0 OR Missing
	me_urinealbumin_prior	Most recent Urine Albumin measurement on/before HF diagnosis	mg/L	0 - 40,000
	me_urinealbumin_prior_lag	Lag between 'me_urinealbumin_prior' and HF diagnosis	days	≥ 0 OR Missing
	me_urinealbumin	Most recent Urine Albumin measurement on/before current observation date	mg/L	0 - 40,000
	me_urinealbumin_lag	Lag between 'me_urinealbumin' and current observation date	days	≥ 0 OR Missing
	me_acr_prior	Most recent Albumin:Creatinine Ratio (ACR) measurement on/before HF diagnosis	mg/mmol	0 - 4000
	me_acr_prior_lag	Lag between 'me_acr_prior' and HF diagnosis	days	≥ 0 OR Missing
	me_acr	Most recent Albumin:Creatinine Ratio (ACR) measurement on/before current observation date	mg/mmol	0 - 4000
	me_acr_lag	Lag between 'me_acr' and current observation date	days	≥ 0 OR Missing
Chronic comorbidities	co_afib_dfirst	Date of first Atrial Fibrillation diagnostic record	date	DD-MM-YYYY
	co_afib	Atrial Fibrillation indicator	binary	0,1
	co_afib_dur	Duration with Atrial Fibrillation up to current observation date	days	≥ 0
	co_cardiomyop_dfirst	Date of first Cardiomyopathy diagnostic record	date	DD-MM-YYYY
	co_cardiomyop	Cardiomyopathy indicator	binary	0,1
	co_cardiomyop_dur	Duration with Cardiomyopathy up to current observation date	days	≥ 0
	co_liver_disease_dfirst	Date of first Chronic Liver Disease diagnostic record	date	DD-MM-YYYY
	co_liver_disease	Chronic Liver Disease indicator	binary	0,1
	co_liver_disease_dur	Duration with Chronic Liver Disease up to current observation date	days	≥ 0
	co_diab_t1_dfirst	Date of first Type 1 Diabetes diagnostic record	date	DD-MM-YYYY

	co_diab_t1	Type 1 Diabetes indicator	binary	0,1
	co_diab_t1_dur	Duration with Type 1 Diabetes up to current observation date	days	≥ 0
	co_diab_t2_dfirst	Date of first Type 2 Diabetes diagnostic record	date	DD-MM-YYYY
	co_diab_t2	Type 2 Diabetes indicator	binary	0,1
	co_diab_t2_dur	Duration with Type 2 Diabetes up to current observation date	days	≥ 0
	co_diab_unspec_dfirst	Date of first Unspecified Diabetes diagnostic record	date	DD-MM-YYYY
	co_diab_unspec	Unspecified Diabetes indicator	binary	0,1
	co_diab_unspec_dur	Duration with Unspecified Diabetes up to current observation date	days	≥ 0
	co_diab_any_dfirst	Date of first Any Diabetes diagnostic record	date	DD-MM-YYYY
	co_diab_any	Any Diabetes indicator	binary	0,1
	co_diab_any_dur	Duration with Any Diabetes up to current observation date	days	≥ 0
	co_glomer_dfirst	Date of first Glomerulonephritis diagnostic record	date	DD-MM-YYYY
	co_glomer	Glomerulonephritis indicator	binary	0,1
	co_glomer_dur	Duration with Glomerulonephritis up to current observation date	days	≥ 0
	co_ischaemic_dfirst	Date of first Ischaemic Heart Disease diagnostic record	date	DD-MM-YYYY
	co_ischaemic	Ischaemic Heart Disease indicator	binary	0,1
	co_ischaemic_dur	Duration with Ischaemic Heart Disease up to current observation date	days	≥ 0
	co_lupus_dfirst	Date of first Systemic Lupus Erythematosus (SLE) diagnostic record	date	DD-MM-YYYY
	co_lupus	Systemic Lupus Erythematosus (SLE) indicator	binary	0,1
	co_lupus_dur	Duration with Systemic Lupus Erythematosus (SLE) up to current observation date	days	≥ 0
	co_nephritis_dfirst	Date of first Nephritis diagnostic record	date	DD-MM-YYYY
	co_nephritis	Nephritis indicator	binary	0,1
	co_nephritis_dur	Duration with Nephritis up to current observation date	days	≥ 0
	co_proteinuria_dfirst	Date of first Proteinuria diagnostic record	date	DD-MM-YYYY
	co_proteinuria	Proteinuria indicator	binary	0,1
	co_proteinuria_dur	Duration with Proteinuria up to current observation date	days	≥ 0
	co_pvd_dfirst	Date of first Peripheral Vascular Disease (PVD) diagnostic record	date	DD-MM-YYYY
	co_pvd	Peripheral Vascular Disease (PVD) indicator	binary	0,1
	co_pvd_dur	Duration with Peripheral Vascular Disease (PVD) up to current observation date	days	≥ 0
	co_valvular_dfirst	Date of first Valvular Heart Disease diagnostic record	date	DD-MM-YYYY
	co_valvular	Valvular Heart Disease indicator	binary	0,1
	co_valvular_dur	Duration with Valvular Heart Disease up to current observation date	days	≥ 0
	co_ventric_hypertr_dfirst	Date of first Ventricular Hypertrophy diagnostic record	date	DD-MM-YYYY
	co_ventric_hypertr	Ventricular Hypertrophy indicator	binary	0,1
	co_ventric_hypertr_dur	Duration with Ventricular Hypertrophy up to current observation date	days	≥ 0
Historical events/ conditions	ev_nephrect_dfirst	Date of first Nephrectomy record	date	DD-MM-YYYY
	ev_nephrect_hx	History of Nephrectomy on/before current observation date	binary	0,1
	ev_stones_dfirst	Date of first Kidney Stones record	date	DD-MM-YYYY
	ev_stones_hx	History of Kidney Stones on/before current observation date	binary	0,1
	ev_nephrot_synd_dfirst	Date of first Nephrotic Syndrome record	date	DD-MM-YYYY
	ev_nephrot_synd_hx	History of Nephrotic Syndrome on/before current observation date	binary	0,1
	ev_ventric_tachy_dfirst	Date of first Ventricular Tachycardia record	date	DD-MM-YYYY
	ev_ventric_tachy_hx	History of Ventricular Tachycardia on/before current observation date	binary	0,1
Episodic conditions	ep_hyperten_dfirst	Date of first Hypertension record	date	DD-MM-YYYY
	ep_hyperten_prior	Episode(s) of Hypertension on/before HF diagnosis	binary	0,1
	ep_hyperten	Current episode of Hypertension (on observation date)	binary	0,1
	ep_hyperten_bin	Episode(s) of Hypertension on/before current observation date	binary	0,1
	ep_hyperten_hx	Episode(s) of Hypertension before current observation date	binary	0,1
	ep_hyperten_new	First episode of Hypertension starting on current observation date	binary	0,1
	ep_hyperten_currdur	Duration of current episode of Hypertension	days	≥ 0
	ep_hyperten_totaldur	Total duration with Hypertension on/before current observation date	days	≥ 0
	ep_hyperkal_dfirst	Date of first Hyperkalaemia record	date	DD-MM-YYYY
	ep_hyperkal_prior	Episode(s) of Hyperkalaemia on/before HF diagnosis	binary	0,1
	ep_hyperkal	Current episode of Hyperkalaemia (on observation date)	binary	0,1
	ep_hyperkal_bin	Episode(s) of Hyperkalaemia on/before current observation date	binary	0,1
	ep_hyperkal_hx	Episode(s) of Hyperkalaemia before current observation date	binary	0,1
	ep_hyperkal_new	First episode of Hyperkalaemia starting on current observation date	binary	0,1

	ep_hyperkal_currdur	Duration of current episode of Hyperkalaemia	days	≥ 0
	ep_hyperkal_totaldur	Total duration with Hyperkalaemia on/before current observation date	days	≥ 0
Prescribed medications	dr_antimicrob_prior	Prescribed Specific Antimicrobial before HF diagnosis	binary	0,1
	dr_antimicrob_bin	Prescribed Specific Antimicrobial on/before current observation date	binary	0,1
	dr_antimicrob_cat	Specific Antimicrobial usage status on current observation date	categorical	Never/Past/Recent/Current
	dr_antimicrob_currdur	Duration of current continuous spell of Specific Antimicrobial usage (dr_antimicrob_cat=="Current")	days	> 0 OR Missing
	dr_antimicrob_totaldur	Total duration of Specific Antimicrobial usage on or before current observation date	days	> 0 OR Missing
	dr_antimicrob_statusdur	Duration of current spell (Past/Recent/Current)	days	> 0 OR Missing
	dr_antimicrob_recent_1	Recent initiated first-ever use of Specific Antimicrobial	binary	0,1
	dr_antimicrob_recent	Recent initiated use of Specific Antimicrobial	binary	0,1
	dr_antimicrob_dose	Current dose of Specific Antimicrobial (based on most recent prescription)	continuous	> 0 OR missing
	dr_antimicrob_dose_cat	Categorised (and standardised) dose of Specific Antimicrobial (based on most recent prescription)	categorical	None/Low/High
	dr_antimicrob_dose_cumul	Cumulative dose of Specific Antimicrobial (up to and including most recent prescription)	continuous	> 0 OR missing
	dr_antimicrob_dose_prev	Previous dose of Specific Antimicrobial (based on previous prescription)	continuous	> 0 OR missing
	dr_antimicrob_dose_prev_cat	Categorised (and standardised) previous dose of Specific Antimicrobial (based on previous prescription)	categorical	None/Low/High
	dr_antimicrob_dose_prev_lag	Lag between most recent and previous prescription of Specific Antimicrobial	days	> 0 OR Missing
	dr_antimicrob_dose_titrate	Recently titrated dose of Specific Antimicrobial	categorical	None/Down/Up
	dr_entresto_prior	Prescribed Entresto before HF diagnosis	binary	0,1
	dr_entresto_bin	Prescribed Entresto on/before current observation date	binary	0,1
	dr_entresto_cat	Entresto usage status on current observation date	categorical	Never/Past/Recent/Current
	dr_entresto_currdur	Duration of current continuous spell of Entresto usage (dr_entresto_cat=="Current")	days	> 0 OR Missing
	dr_entresto_totaldur	Total duration of Entresto usage on or before current observation date	days	> 0 OR Missing
	dr_entresto_statusdur	Duration of current spell (Past/Recent/Current)	days	> 0 OR Missing
	dr_entresto_recent_1	Recent initiated first-ever use of Entresto	binary	0,1
	dr_entresto_recent	Recent initiated use of Entresto	binary	0,1
	dr_entresto_dose	Current dose of Entresto (based on most recent prescription)	continuous	> 0 OR missing
	dr_entresto_dose_cat	Categorised (and standardised) dose of Entresto (based on most recent prescription)	categorical	None/Low/High
	dr_entresto_dose_cumul	Cumulative dose of Entresto (up to and including most recent prescription)	continuous	> 0 OR missing
	dr_entresto_dose_prev	Previous dose of Entresto (based on previous prescription)	continuous	> 0 OR missing
	dr_entresto_dose_prev_cat	Categorised (and standardised) previous dose of Entresto (based on previous prescription)	categorical	None/Low/High
	dr_entresto_dose_prev_lag	Lag between most recent and previous prescription of Entresto	days	> 0 OR Missing
	dr_entresto_dose_titrate	Recently titrated dose of Entresto	categorical	None/Down/Up
	dr_ace_prior	Prescribed ACE Inhibitor before HF diagnosis	binary	0,1
	dr_ace_bin	Prescribed ACE Inhibitor on/before current observation date	binary	0,1
	dr_ace_cat	ACE Inhibitor usage status on current observation date	categorical	Never/Past/Recent/Current
	dr_ace_currdur	Duration of current continuous spell of ACE Inhibitor usage (dr_ace_cat=="Current")	days	> 0 OR Missing
	dr_ace_totaldur	Total duration of ACE Inhibitor usage on or before current observation date	days	> 0 OR Missing
	dr_ace_statusdur	Duration of current spell (Past/Recent/Current)	days	> 0 OR Missing
	dr_ace_recent_1	Recent initiated first-ever use of ACE Inhibitor	binary	0,1
	dr_ace_recent	Recent initiated use of ACE Inhibitor	binary	0,1
	dr_ace_dose	Current dose of ACE Inhibitor (based on most recent prescription)	continuous	> 0 OR missing
	dr_ace_dose_cat	Categorised (and standardised) dose of ACE Inhibitor (based on most recent prescription)	categorical	None/Low/High
	dr_ace_dose_cumul	Cumulative dose of ACE Inhibitor (up to and including most recent prescription)	continuous	> 0 OR missing
	dr_ace_dose_prev	Previous dose of ACE Inhibitor (based on previous prescription)	continuous	> 0 OR missing
	dr_ace_dose_prev_cat	Categorised (and standardised) previous dose of ACE Inhibitor (based on previous prescription)	categorical	None/Low/High
	dr_ace_dose_prev_lag	Lag between most recent and previous prescription of ACE Inhibitor	days	> 0 OR Missing
	dr_ace_dose_titrate	Recently titrated dose of ACE Inhibitor	categorical	None/Down/Up
	dr_arb_prior	Prescribed Angiotensin Receptor Blocker (ARB) before HF diagnosis	binary	0,1
	dr_arb_bin	Prescribed Angiotensin Receptor Blocker (ARB) on/before current observation date	binary	0,1
	dr_arb_cat	Angiotensin Receptor Blocker (ARB) usage status on current observation date	categorical	Never/Past/Recent/Current
	dr_arb_currdur	Duration of current continuous spell of Angiotensin Receptor Blocker (ARB) usage (dr_arb_cat=="Current")	days	> 0 OR Missing
	dr_arb_totaldur	Total duration of Angiotensin Receptor Blocker (ARB) usage on or before current observation date	days	> 0 OR Missing
	dr_arb_statusdur	Duration of current spell (Past/Recent/Current)	days	> 0 OR Missing
	dr_arb_recent_1	Recent initiated first-ever use of Angiotensin Receptor Blocker (ARB)	binary	0,1
	dr_arb_recent	Recent initiated use of Angiotensin Receptor Blocker (ARB)	binary	0,1
	dr_arb_dose	Current dose of Angiotensin Receptor Blocker (ARB) (based on most recent prescription)	continuous	> 0 OR missing
	dr_arb_dose_cat	Categorised (and standardised) dose of Angiotensin Receptor Blocker (ARB) (based on most recent prescription)	categorical	None/Low/High

dr_arb_dose_cumul	Cumulative dose of Angiotensin Receptor Blocker (ARB) (up to and including most recent prescription)	continuous	> 0 OR missing
dr_arb_dose_prev	Previous dose of Angiotensin Receptor Blocker (ARB) (based on previous prescription)	continuous	> 0 OR missing
dr_arb_dose_prev_cat	Categorised (and standardised) previous dose of Angiotensin Receptor Blocker (ARB) (based on previous prescription)	categorical	None/Low/High
dr_arb_dose_prev_lag	Lag between most recent and previous prescription of Angiotensin Receptor Blocker (ARB)	days	> 0 OR Missing
dr_arb_dose_titrate	Recently titrated dose of Angiotensin Receptor Blocker (ARB)	categorical	None/Down/Up
dr_alpha_prior	Prescribed Alpha-Blocker before HF diagnosis	binary	0,1
dr_alpha_bin	Prescribed Alpha-Blocker on/before current observation date	binary	0,1
dr_alpha_cat	Alpha-Blocker usage status on current observation date	categorical	Never/Past/Recent/Current
dr_alpha_currdur	Duration of current continuous spell of Alpha-Blocker usage (dr_alpha_cat=="Current")	days	> 0 OR Missing
dr_alpha_totaldur	Total duration of Alpha-Blocker usage on or before current observation date	days	> 0 OR Missing
dr_alpha_statusdur	Duration of current spell (Past/Recent/Current)	days	> 0 OR Missing
dr_alpha_recent_1	Recent initiated first-ever use of Alpha-Blocker	binary	0,1
dr_alpha_recent	Recent initiated use of Alpha-Blocker	binary	0,1
dr_alpha_dose	Current dose of Alpha-Blocker (based on most recent prescription)	continuous	> 0 OR missing
dr_alpha_dose_cat	Categorised (and standardised) dose of Alpha-Blocker (based on most recent prescription)	categorical	None/Low/High
dr_alpha_dose_cumul	Cumulative dose of Alpha-Blocker (up to and including most recent prescription)	continuous	> 0 OR missing
dr_alpha_dose_prev	Previous dose of Alpha-Blocker (based on previous prescription)	continuous	> 0 OR missing
dr_alpha_dose_prev_cat	Categorised (and standardised) previous dose of Alpha-Blocker (based on previous prescription)	categorical	None/Low/High
dr_alpha_dose_prev_lag	Lag between most recent and previous prescription of Alpha-Blocker	days	> 0 OR Missing
dr_alpha_dose_titrate	Recently titrated dose of Alpha-Blocker	categorical	None/Down/Up
dr_beta_prior	Prescribed Beta-Blocker before HF diagnosis	binary	0,1
dr_beta_bin	Prescribed Beta-Blocker on/before current observation date	binary	0,1
dr_beta_cat	Beta-Blocker usage status on current observation date	categorical	Never/Past/Recent/Current
dr_beta_currdur	Duration of current continuous spell of Beta-Blocker usage (dr_beta_cat=="Current")	days	> 0 OR Missing
dr_beta_totaldur	Total duration of Beta-Blocker usage on or before current observation date	days	> 0 OR Missing
dr_beta_statusdur	Duration of current spell (Past/Recent/Current)	days	> 0 OR Missing
dr_beta_recent_1	Recent initiated first-ever use of Beta-Blocker	binary	0,1
dr_beta_recent	Recent initiated use of Beta-Blocker	binary	0,1
dr_beta_dose	Current dose of Beta-Blocker (based on most recent prescription)	continuous	> 0 OR missing
dr_beta_dose_cat	Categorised (and standardised) dose of Beta-Blocker (based on most recent prescription)	categorical	None/Low/High
dr_beta_dose_cumul	Cumulative dose of Beta-Blocker (up to and including most recent prescription)	continuous	> 0 OR missing
dr_beta_dose_prev	Previous dose of Beta-Blocker (based on previous prescription)	continuous	> 0 OR missing
dr_beta_dose_prev_cat	Categorised (and standardised) previous dose of Beta-Blocker (based on previous prescription)	categorical	None/Low/High
dr_beta_dose_prev_lag	Lag between most recent and previous prescription of Beta-Blocker	days	> 0 OR Missing
dr_beta_dose_titrate	Recently titrated dose of Beta-Blocker	categorical	None/Down/Up
dr_calciumch_prior	Prescribed Calcium Channel Blocker before HF diagnosis	binary	0,1
dr_calciumch_bin	Prescribed Calcium Channel Blocker on/before current observation date	binary	0,1
dr_calciumch_cat	Calcium Channel Blocker usage status on current observation date	categorical	Never/Past/Recent/Current
dr_calciumch_currdur	Duration of current continuous spell of Calcium Channel Blocker usage (dr_calciumch_cat=="Current")	days	> 0 OR Missing
dr_calciumch_totaldur	Total duration of Calcium Channel Blocker usage on or before current observation date	days	> 0 OR Missing
dr_calciumch_statusdur	Duration of current spell (Past/Recent/Current)	days	> 0 OR Missing
dr_calciumch_recent_1	Recent initiated first-ever use of Calcium Channel Blocker	binary	0,1
dr_calciumch_recent	Recent initiated use of Calcium Channel Blocker	binary	0,1
dr_calciumch_dose	Current dose of Calcium Channel Blocker (based on most recent prescription)	continuous	> 0 OR missing
dr_calciumch_dose_cat	Categorised (and standardised) dose of Calcium Channel Blocker (based on most recent prescription)	categorical	None/Low/High
dr_calciumch_dose_cumul	Cumulative dose of Calcium Channel Blocker (up to and including most recent prescription)	continuous	> 0 OR missing
dr_calciumch_dose_prev	Previous dose of Calcium Channel Blocker (based on previous prescription)	continuous	> 0 OR missing
dr_calciumch_dose_prev_cat	Categorised (and standardised) previous dose of Calcium Channel Blocker (based on previous prescription)	categorical	None/Low/High
dr_calciumch_dose_prev_lag	Lag between most recent and previous prescription of Calcium Channel Blocker	days	> 0 OR Missing
dr_calciumch_dose_titrate	Recently titrated dose of Calcium Channel Blocker	categorical	None/Down/Up
dr_central_prior	Prescribed Central-Acting Agent before HF diagnosis	binary	0,1
dr_central_bin	Prescribed Central-Acting Agent on/before current observation date	binary	0,1
dr_central_cat	Central-Acting Agent usage status on current observation date	categorical	Never/Past/Recent/Current
dr_central_currdur	Duration of current continuous spell of Central-Acting Agent usage (dr_central_cat=="Current")	days	> 0 OR Missing
dr_central_totaldur	Total duration of Central-Acting Agent usage on or before current observation date	days	> 0 OR Missing
dr_central_statusdur	Duration of current spell (Past/Recent/Current)	days	> 0 OR Missing
dr_central_recent_1	Recent initiated first-ever use of Central-Acting Agent	binary	0,1

	dr_central_recent	Recent initiated use of Central-Acting Agent	binary	0,1
	dr_central_dose	Current dose of Central-Acting Agent (based on most recent prescription)	continuous	> 0 OR missing
	dr_central_dose_cat	Categorised (and standardised) dose of Central-Acting Agent (based on most recent prescription)	categorical	None/Low/High
	dr_central_dose_cumul	Cumulative dose of Central-Acting Agent (up to and including most recent prescription)	continuous	> 0 OR missing
	dr_central_dose_prev	Previous dose of Central-Acting Agent (based on previous prescription)	continuous	> 0 OR missing
	dr_central_dose_prev_cat	Categorised (and standardised) previous dose of Central-Acting Agent (based on previous prescription)	categorical	None/Low/High
	dr_central_dose_prev_lag	Lag between most recent and previous prescription of Central-Acting Agent	days	> 0 OR Missing
	dr_central_dose_titrate	Recently titrated dose of Central-Acting Agent	categorical	None/Down/Up
	dr_renin_prior	Prescribed Direct Renin Inhibitor before HF diagnosis	binary	0,1
	dr_renin_bin	Prescribed Direct Renin Inhibitor on/before current observation date	binary	0,1
	dr_renin_cat	Direct Renin Inhibitor usage status on current observation date	categorical	Never/Past/Recent/Current
	dr_renin_currdur	Duration of current continuous spell of Direct Renin Inhibitor usage (dr_renin_cat=="Current")	days	> 0 OR Missing
	dr_renin_totaldur	Total duration of Direct Renin Inhibitor usage on or before current observation date	days	> 0 OR Missing
	dr_renin_statusdur	Duration of current spell (Past/Recent/Current)	days	> 0 OR Missing
	dr_renin_recent_1	Recent initiated first-ever use of Direct Renin Inhibitor	binary	0,1
	dr_renin_recent	Recent initiated use of Direct Renin Inhibitor	binary	0,1
	dr_renin_dose	Current dose of Direct Renin Inhibitor (based on most recent prescription)	continuous	> 0 OR missing
	dr_renin_dose_cat	Categorised (and standardised) dose of Direct Renin Inhibitor (based on most recent prescription)	categorical	None/Low/High
	dr_renin_dose_cumul	Cumulative dose of Direct Renin Inhibitor (up to and including most recent prescription)	continuous	> 0 OR missing
	dr_renin_dose_prev	Previous dose of Direct Renin Inhibitor (based on previous prescription)	continuous	> 0 OR missing
	dr_renin_dose_prev_cat	Categorised (and standardised) previous dose of Direct Renin Inhibitor (based on previous prescription)	categorical	None/Low/High
	dr_renin_dose_prev_lag	Lag between most recent and previous prescription of Direct Renin Inhibitor	days	> 0 OR Missing
	dr_renin_dose_titrate	Recently titrated dose of Direct Renin Inhibitor	categorical	None/Down/Up
	dr_loop_prior	Prescribed Loop Diuretic before HF diagnosis	binary	0,1
	dr_loop_bin	Prescribed Loop Diuretic on/before current observation date	binary	0,1
	dr_loop_cat	Loop Diuretic usage status on current observation date	categorical	Never/Past/Recent/Current
	dr_loop_currdur	Duration of current continuous spell of Loop Diuretic usage (dr_loop_cat=="Current")	days	> 0 OR Missing
	dr_loop_totaldur	Total duration of Loop Diuretic usage on or before current observation date	days	> 0 OR Missing
	dr_loop_statusdur	Duration of current spell (Past/Recent/Current)	days	> 0 OR Missing
	dr_loop_recent_1	Recent initiated first-ever use of Loop Diuretic	binary	0,1
	dr_loop_recent	Recent initiated use of Loop Diuretic	binary	0,1
	dr_loop_dose	Current dose of Loop Diuretic (based on most recent prescription)	continuous	> 0 OR missing
	dr_loop_dose_cat	Categorised (and standardised) dose of Loop Diuretic (based on most recent prescription)	categorical	None/Low/High
	dr_loop_dose_cumul	Cumulative dose of Loop Diuretic (up to and including most recent prescription)	continuous	> 0 OR missing
	dr_loop_dose_prev	Previous dose of Loop Diuretic (based on previous prescription)	continuous	> 0 OR missing
	dr_loop_dose_prev_cat	Categorised (and standardised) previous dose of Loop Diuretic (based on previous prescription)	categorical	None/Low/High
	dr_loop_dose_prev_lag	Lag between most recent and previous prescription of Loop Diuretic	days	> 0 OR Missing
	dr_loop_dose_titrate	Recently titrated dose of Loop Diuretic	categorical	None/Down/Up
	dr_pai_prior	Prescribed Peripheral Adrenergic Inhibitor before HF diagnosis	binary	0,1
	dr_pai_bin	Prescribed Peripheral Adrenergic Inhibitor on/before current observation date	binary	0,1
	dr_pai_cat	Peripheral Adrenergic Inhibitor usage status on current observation date	categorical	Never/Past/Recent/Current
	dr_pai_currdur	Duration of current continuous spell of Peripheral Adrenergic Inhibitor usage (dr_pai_cat=="Current")	days	> 0 OR Missing
	dr_pai_totaldur	Total duration of Peripheral Adrenergic Inhibitor usage on or before current observation date	days	> 0 OR Missing
	dr_pai_statusdur	Duration of current spell (Past/Recent/Current)	days	> 0 OR Missing
	dr_pai_recent_1	Recent initiated first-ever use of Peripheral Adrenergic Inhibitor	binary	0,1
	dr_pai_recent	Recent initiated use of Peripheral Adrenergic Inhibitor	binary	0,1
	dr_pai_dose	Current dose of Peripheral Adrenergic Inhibitor (based on most recent prescription)	continuous	> 0 OR missing
	dr_pai_dose_cat	Categorised (and standardised) dose of Peripheral Adrenergic Inhibitor (based on most recent prescription)	categorical	None/Low/High
	dr_pai_dose_cumul	Cumulative dose of Peripheral Adrenergic Inhibitor (up to and including most recent prescription)	continuous	> 0 OR missing
	dr_pai_dose_prev	Previous dose of Peripheral Adrenergic Inhibitor (based on previous prescription)	continuous	> 0 OR missing
	dr_pai_dose_prev_cat	Categorised (and standardised) previous dose of Peripheral Adrenergic Inhibitor (based on previous prescription)	categorical	None/Low/High
	dr_pai_dose_prev_lag	Lag between most recent and previous prescription of Peripheral Adrenergic Inhibitor	days	> 0 OR Missing
	dr_pai_dose_titrate	Recently titrated dose of Peripheral Adrenergic Inhibitor	categorical	None/Down/Up
	dr_potass_prior	Prescribed Potassium-Sparing Diuretic before HF diagnosis	binary	0,1
	dr_potass_bin	Prescribed Potassium-Sparing Diuretic on/before current observation date	binary	0,1
	dr_potass_cat	Potassium-Sparing Diuretic usage status on current observation date	categorical	Never/Past/Recent/Current
	dr_potass_currdur	Duration of current continuous spell of Potassium-Sparing Diuretic usage (dr_potass_cat=="Current")	days	> 0 OR Missing

	dr_potass_totaldur	Total duration of Potassium-Sparing Diuretic usage on or before current observation date	days	> 0 OR Missing
	dr_potass_statusdur	Duration of current spell (Past/Recent/Current)	days	> 0 OR Missing
	dr_potass_recent_1	Recent initiated first-ever use of Potassium-Sparing Diuretic	binary	0,1
	dr_potass_recent	Recent initiated use of Potassium-Sparing Diuretic	binary	0,1
	dr_potass_dose	Current dose of Potassium-Sparing Diuretic (based on most recent prescription)	continuous	> 0 OR missing
	dr_potass_dose_cat	Categorised (and standardised) dose of Potassium-Sparing Diuretic (based on most recent prescription)	categorical	None/Low/High
	dr_potass_dose_cumul	Cumulative dose of Potassium-Sparing Diuretic (up to and including most recent prescription)	continuous	> 0 OR missing
	dr_potass_dose_prev	Previous dose of Potassium-Sparing Diuretic (based on previous prescription)	continuous	> 0 OR missing
	dr_potass_dose_prev_cat	Categorised (and standardised) previous dose of Potassium-Sparing Diuretic (based on previous prescription)	categorical	None/Low/High
	dr_potass_dose_prev_lag	Lag between most recent and previous prescription of Potassium-Sparing Diuretic	days	> 0 OR Missing
	dr_potass_dose_titrate	Recently titrated dose of Potassium-Sparing Diuretic	categorical	None/Down/Up
	dr_thiaz_prior	Prescribed Thiazide Diuretic before HF diagnosis	binary	0,1
	dr_thiaz_bin	Prescribed Thiazide Diuretic on/before current observation date	binary	0,1
	dr_thiaz_cat	Thiazide Diuretic usage status on current observation date	categorical	Never/Past/Recent/Current
	dr_thiaz_currdur	Duration of current continuous spell of Thiazide Diuretic usage (dr_thiaz_cat=="Current")	days	> 0 OR Missing
	dr_thiaz_totaldur	Total duration of Thiazide Diuretic usage on or before current observation date	days	> 0 OR Missing
	dr_thiaz_statusdur	Duration of current spell (Past/Recent/Current)	days	> 0 OR Missing
	dr_thiaz_recent_1	Recent initiated first-ever use of Thiazide Diuretic	binary	0,1
	dr_thiaz_recent	Recent initiated use of Thiazide Diuretic	binary	0,1
	dr_thiaz_dose	Current dose of Thiazide Diuretic (based on most recent prescription)	continuous	> 0 OR missing
	dr_thiaz_dose_cat	Categorised (and standardised) dose of Thiazide Diuretic (based on most recent prescription)	categorical	None/Low/High
	dr_thiaz_dose_cumul	Cumulative dose of Thiazide Diuretic (up to and including most recent prescription)	continuous	> 0 OR missing
	dr_thiaz_dose_prev	Previous dose of Thiazide Diuretic (based on previous prescription)	continuous	> 0 OR missing
	dr_thiaz_dose_prev_cat	Categorised (and standardised) previous dose of Thiazide Diuretic (based on previous prescription)	categorical	None/Low/High
	dr_thiaz_dose_prev_lag	Lag between most recent and previous prescription of Thiazide Diuretic	days	> 0 OR Missing
	dr_thiaz_dose_titrate	Recently titrated dose of Thiazide Diuretic	categorical	None/Down/Up
	dr_vasodil_prior	Prescribed Vasodilator before HF diagnosis	binary	0,1
	dr_vasodil_bin	Prescribed Vasodilator on/before current observation date	binary	0,1
	dr_vasodil_cat	Vasodilator usage status on current observation date	categorical	Never/Past/Recent/Current
	dr_vasodil_currdur	Duration of current continuous spell of Vasodilator usage (dr_vasodil_cat=="Current")	days	> 0 OR Missing
	dr_vasodil_totaldur	Total duration of Vasodilator usage on or before current observation date	days	> 0 OR Missing
	dr_vasodil_statusdur	Duration of current spell (Past/Recent/Current)	days	> 0 OR Missing
	dr_vasodil_recent_1	Recent initiated first-ever use of Vasodilator	binary	0,1
	dr_vasodil_recent	Recent initiated use of Vasodilator	binary	0,1
	dr_vasodil_dose	Current dose of Vasodilator (based on most recent prescription)	continuous	> 0 OR missing
	dr_vasodil_dose_cat	Categorised (and standardised) dose of Vasodilator (based on most recent prescription)	categorical	None/Low/High
	dr_vasodil_dose_cumul	Cumulative dose of Vasodilator (up to and including most recent prescription)	continuous	> 0 OR missing
	dr_vasodil_dose_prev	Previous dose of Vasodilator (based on previous prescription)	continuous	> 0 OR missing
	dr_vasodil_dose_prev_cat	Categorised (and standardised) previous dose of Vasodilator (based on previous prescription)	categorical	None/Low/High
	dr_vasodil_dose_prev_lag	Lag between most recent and previous prescription of Vasodilator	days	> 0 OR Missing
	dr_vasodil_dose_titrate	Recently titrated dose of Vasodilator	categorical	None/Down/Up
	dr_immuno_prior	Prescribed Specific Immunosuppressant before HF diagnosis	binary	0,1
	dr_immuno_bin	Prescribed Specific Immunosuppressant on/before current observation date	binary	0,1
	dr_immuno_cat	Specific Immunosuppressant usage status on current observation date	categorical	Never/Past/Recent/Current
	dr_immuno_currdur	Duration of current continuous spell of Specific Immunosuppressant usage (dr_immuno_cat=="Current")	days	> 0 OR Missing
	dr_immuno_totaldur	Total duration of Specific Immunosuppressant usage on or before current observation date	days	> 0 OR Missing
	dr_immuno_statusdur	Duration of current spell (Past/Recent/Current)	days	> 0 OR Missing
	dr_immuno_recent_1	Recent initiated first-ever use of Specific Immunosuppressant	binary	0,1
	dr_immuno_recent	Recent initiated use of Specific Immunosuppressant	binary	0,1
	dr_immuno_dose	Current dose of Specific Immunosuppressant (based on most recent prescription)	continuous	> 0 OR missing
	dr_immuno_dose_cat	Categorised (and standardised) dose of Specific Immunosuppressant (based on most recent prescription)	categorical	None/Low/High
	dr_immuno_dose_cumul	Cumulative dose of Specific Immunosuppressant (up to and including most recent prescription)	continuous	> 0 OR missing
	dr_immuno_dose_prev	Previous dose of Specific Immunosuppressant (based on previous prescription)	continuous	> 0 OR missing
	dr_immuno_dose_prev_cat	Categorised (and standardised) previous dose of Specific Immunosuppressant (based on previous prescription)	categorical	None/Low/High
	dr_immuno_dose_prev_lag	Lag between most recent and previous prescription of Specific Immunosuppressant	days	> 0 OR Missing
	dr_immuno_dose_titrate	Recently titrated dose of Specific Immunosuppressant	categorical	None/Down/Up
	dr_nsaid_prior	Prescribed NSAID before HF diagnosis	binary	0,1

	dr_nsaid_bin	Prescribed NSAID on/before current observation date	binary	0,1
	dr_nsaid_cat	NSAID usage status on current observation date	categorical	Never/Past/Recent/Current
	dr_nsaid_currdur	Duration of current continuous spell of NSAID usage (dr_nsaid_cat=="Current")	days	> 0 OR Missing
	dr_nsaid_totaldur	Total duration of NSAID usage on or before current observation date	days	> 0 OR Missing
	dr_nsaid_statusdur	Duration of current spell (Past/Recent/Current)	days	> 0 OR Missing
	dr_nsaid_recent_1	Recent initiated first-ever use of NSAID	binary	0,1
	dr_nsaid_recent	Recent initiated use of NSAID	binary	0,1
	dr_nsaid_dose	Current dose of NSAID (based on most recent prescription)	continuous	> 0 OR missing
	dr_nsaid_dose_cat	Categorised (and standardised) dose of NSAID (based on most recent prescription)	categorical	None/Low/High
	dr_nsaid_dose_cumul	Cumulative dose of NSAID (up to and including most recent prescription)	continuous	> 0 OR missing
	dr_nsaid_dose_prev	Previous dose of NSAID (based on previous prescription)	continuous	> 0 OR missing
	dr_nsaid_dose_prev_cat	Categorised (and standardised) previous dose of NSAID (based on previous prescription)	categorical	None/Low/High
	dr_nsaid_dose_prev_lag	Lag between most recent and previous prescription of NSAID	days	> 0 OR Missing
	dr_nsaid_dose_titrate	Recently titrated dose of NSAID	categorical	None/Down/Up
	dr_proton_prior	Prescribed Proton-Pump Inhibitor before HF diagnosis	binary	0,1
	dr_proton_bin	Prescribed Proton-Pump Inhibitor on/before current observation date	binary	0,1
	dr_proton_cat	Proton-Pump Inhibitor usage status on current observation date	categorical	Never/Past/Recent/Current
	dr_proton_currdur	Duration of current continuous spell of Proton-Pump Inhibitor usage (dr_proton_cat=="Current")	days	> 0 OR Missing
	dr_proton_totaldur	Total duration of Proton-Pump Inhibitor usage on or before current observation date	days	> 0 OR Missing
	dr_proton_statusdur	Duration of current spell (Past/Recent/Current)	days	> 0 OR Missing
	dr_proton_recent_1	Recent initiated first-ever use of Proton-Pump Inhibitor	binary	0,1
	dr_proton_recent	Recent initiated use of Proton-Pump Inhibitor	binary	0,1
	dr_proton_dose	Current dose of Proton-Pump Inhibitor (based on most recent prescription)	continuous	> 0 OR missing
	dr_proton_dose_cat	Categorised (and standardised) dose of Proton-Pump Inhibitor (based on most recent prescription)	categorical	None/Low/High
	dr_proton_dose_cumul	Cumulative dose of Proton-Pump Inhibitor (up to and including most recent prescription)	continuous	> 0 OR missing
	dr_proton_dose_prev	Previous dose of Proton-Pump Inhibitor (based on previous prescription)	continuous	> 0 OR missing
	dr_proton_dose_prev_cat	Categorised (and standardised) previous dose of Proton-Pump Inhibitor (based on previous prescription)	categorical	None/Low/High
	dr_proton_dose_prev_lag	Lag between most recent and previous prescription of Proton-Pump Inhibitor	days	> 0 OR Missing
	dr_proton_dose_titrate	Recently titrated dose of Proton-Pump Inhibitor	categorical	None/Down/Up
	dr_sglt2i_prior	Prescribed SGLT2 Inhibitor before HF diagnosis	binary	0,1
	dr_sglt2i_bin	Prescribed SGLT2 Inhibitor on/before current observation date	binary	0,1
	dr_sglt2i_cat	SGLT2 Inhibitor usage status on current observation date	categorical	Never/Past/Recent/Current
	dr_sglt2i_currdur	Duration of current continuous spell of SGLT2 Inhibitor usage (dr_sglt2i_cat=="Current")	days	> 0 OR Missing
	dr_sglt2i_totaldur	Total duration of SGLT2 Inhibitor usage on or before current observation date	days	> 0 OR Missing
	dr_sglt2i_statusdur	Duration of current spell (Past/Recent/Current)	days	> 0 OR Missing
	dr_sglt2i_recent_1	Recent initiated first-ever use of SGLT2 Inhibitor	binary	0,1
	dr_sglt2i_recent	Recent initiated use of SGLT2 Inhibitor	binary	0,1
	dr_sglt2i_dose	Current dose of SGLT2 Inhibitor (based on most recent prescription)	continuous	> 0 OR missing
	dr_sglt2i_dose_cat	Categorised (and standardised) dose of SGLT2 Inhibitor (based on most recent prescription)	categorical	None/Low/High
	dr_sglt2i_dose_cumul	Cumulative dose of SGLT2 Inhibitor (up to and including most recent prescription)	continuous	> 0 OR missing
	dr_sglt2i_dose_prev	Previous dose of SGLT2 Inhibitor (based on previous prescription)	continuous	> 0 OR missing
	dr_sglt2i_dose_prev_cat	Categorised (and standardised) previous dose of SGLT2 Inhibitor (based on previous prescription)	categorical	None/Low/High
	dr_sglt2i_dose_prev_lag	Lag between most recent and previous prescription of SGLT2 Inhibitor	days	> 0 OR Missing
	dr_sglt2i_dose_titrate	Recently titrated dose of SGLT2 Inhibitor	categorical	None/Down/Up
