# Supplementary material for: Development and validation of clinical prediction models for personalized renal function monitoring in people with heart failure in primary care: the RENAL-HF study protocol
Source: Eur Heart J Digit Health. 2026 Mar 31;7(4):ztag055. doi: 10.1093/ehjdh/ztag055 (PMC13131987; doi:10.1093/ehjdh/ztag055)
Supplement: ztag055_Supplementary_Data [file ztag055_supplementary_data.zip › S3_updated.pdf]

Supplementary File 3: Specification, prediction, assessment, and scheduling in a joint regression modelling framework

1. Specification

Within a joint regression modelling framework, we plan to optimise the estimation of a longitudinal submodel for the observed serum creatinine measurements and a survival submodel for the competing secondary outcomes of hospitalisation due to AKI, MACE, and all-cause mortality.

1.1. Longitudinal submodel

Let $y(s)$ represent the natural logarithm of a given patient's serum creatinine level over time, $s \geq 0$, where the origin is the date of the patient's heart failure diagnosis. We observe $y(s)$ at discrete time points, $t_1 < t_2 <\ldots$ where t_1 is the time of the first measurement on or after diagnosis. Considering M patients in the development/training data, we define the patient index: $i = 1, 2, \ldots, M$ and the observation index: $j = 1, 2, \ldots, n_i$, where n_i is the total number of serum creatinine observation points for the ith patient.

Notation at the observation-level

At time t_{ij}, the jth log serum creatinine measurement for the ith patient is represented as

$$y_{ij} = \mathbf{x}_{ij}'\boldsymbol{\beta} + \mathbf{z}_{ij}'\mathbf{u}_i + \epsilon_{ij} \qquad [1]$$

where $y_{ij} = y(t_{ij})$ is the response (i.e., the log serum creatinine value) at time t_{ij}, $\mathbf{x}_{ij}$ is a $p \times 1$ covariate vector for the $p \times 1$ fixed effects coefficient vector $\boldsymbol{\beta}$, $\mathbf{z}_{ij}$ is the $q \times 1$ design vector for the patient-specific $q \times 1$ random effects vector $\mathbf{u}_i$, and ϵ_{ij} is a mean-zero normally distributed random error term.

Growth curves

The design vectors $\mathbf{x}_{ij}$ and $\mathbf{z}_{ij}$ will incorporate functions of time. We will consider polynomial functions, fractional polynomials, structural growth models, and components of linearised versions of non-linear functions, e.g., via Taylor expansions. Interactions between time-based trend components and other covariates/predictors will also be investigated.

Notation at the patient-level

Grouping over observations at the patient-level, we have

$$\mathbf{y}_i = \mathbf{X}_i\boldsymbol{\beta} + \mathbf{Z}_i\mathbf{u}_i + \boldsymbol{\epsilon}_i \qquad [2]$$

where, $\mathbf{y}_i$ is the $n_i \times 1$ response vector $\left(y_{i1}, y_{i2}, \ldots, y_{in_i}\right)'$, $\mathbf{X}_i$ is the $n_i \times p$ covariate matrix $\left(\mathbf{x}_{i1}', \mathbf{x}_{i2}', \ldots, \mathbf{x}_{in_i}'\right)'$, $\mathbf{Z}_i$ is the $n_i \times q$ design matrix $\left(\mathbf{z}_{i1}', \mathbf{z}_{i2}', \ldots, \mathbf{z}_{in_i}'\right)'$, the $q \times 1$ vector of random effects is distributed as $\mathbf{u}_i \sim N(\mathbf{0}, \boldsymbol{\Sigma})$ and $\boldsymbol{\Sigma}$ is a $q \times q$ variance-covariance matrix.

Notation at the cohort-level

Grouping over all patients in the cohort, we have

$$\mathbf{y} = \mathbf{X}\boldsymbol{\beta} + \mathbf{Z}\mathbf{u} + \boldsymbol{\epsilon} \quad [3]$$

where, $\mathbf{y}$ is the $n \times 1$ response vector with $n = \sum_{i=1}^{M} n_i$, $\mathbf{X}$ is an $n \times p$ covariate matrix, $\mathbf{Z}$ is an $n \times Mq$ block-diagonal design matrix with structure

$$\mathbf{Z} = \begin{pmatrix} \mathbf{Z}_1 & \mathbf{0} & \cdots & \mathbf{0} \\ \mathbf{0} & \mathbf{Z}_2 & \cdots & \mathbf{0} \\ \vdots & \vdots & \ddots & \vdots \\ \mathbf{0} & \mathbf{0} & \cdots & \mathbf{Z}_M \end{pmatrix},$$

$\mathbf{u}$ is an $Mq \times 1$ vector of random effects $(\mathbf{u}_1, \mathbf{u}_2, \dots, \mathbf{u}_M)'$ with variance-covariance matrix $\mathbf{G}$, and the $n \times 1$ vector of errors is assumed to be distributed as $\boldsymbol{\epsilon} \sim N(\mathbf{0}, \sigma_\epsilon^2 \mathbf{R})$. Specification of specific structures for $\mathbf{G}$ allow the random intercepts and coefficients to be modelled as independent or correlated. Assuming that the random effects and residual errors are orthogonal, the combined variance-covariance matrix is

$$\text{Var}\begin{bmatrix} \mathbf{u} \\ \boldsymbol{\epsilon} \end{bmatrix} = \begin{bmatrix} \mathbf{G} & \mathbf{0} \\ \mathbf{0} & \sigma_\epsilon^2 \mathbf{R} \end{bmatrix}$$

where σ_ϵ^2 is the overall residual variance, $\mathbf{G} = \mathbf{I}_M \otimes \boldsymbol{\Sigma}$, and $\mathbf{R} = \mathbf{I}_M \otimes \boldsymbol{\Lambda}$ with the simplest form for $\boldsymbol{\Lambda}$ being the identity matrix $\mathbf{I}_{n_i}$ (an assumption we may subsequently relax to consider the potential for heteroskedastic residual errors).

Variable selection

Combining the random effects and residual error terms in (3) as $\mathbf{Z}\mathbf{u} + \boldsymbol{\epsilon}$, the distribution of the response vector $\mathbf{y}$ is multivariate normal with mean $\mathbf{X}\boldsymbol{\beta}$ and $n \times n$ variance–covariance matrix

$$\mathbf{V} = \mathbf{Z}\mathbf{G}\mathbf{Z}' + \sigma_\epsilon^2 \, \mathbf{I}_n \quad [4]$$

The log-likelihood can be written as

$$l(\boldsymbol{\beta}, \boldsymbol{\theta}, \sigma_\epsilon^2) = -\frac{1}{2}\{n \log(2\pi) + \log|\mathbf{V}| + (\mathbf{y} - \mathbf{X}\boldsymbol{\beta})' \, \mathbf{V}^{-1}(\mathbf{y} - \mathbf{X}\boldsymbol{\beta})\} \quad [5]$$

which is maximized as a function of $\boldsymbol{\beta}$, $\boldsymbol{\theta}$ (a vector containing the unique elements in $\mathbf{G}$), and σ_ϵ^2. For variable selection, we will use the Least Absolute Shrinkage and Selection Operator (LASSO) proposed by Tibshirani (1996) which is based on penalised regression and uses an L_1-penalty on the regression coefficients. The log-likelihood $l(\boldsymbol{\beta}, \boldsymbol{\theta}, \sigma_\epsilon^2)$ is maximised while constraining the L_1-norm of the parameter vector $\boldsymbol{\beta}$. The LASSO estimate $\widehat{\boldsymbol{\beta}}$ is obtained as the solution to

$$\widehat{\boldsymbol{\beta}} = \underset{\boldsymbol{\beta}}{\text{argmax}}[l(\boldsymbol{\beta}, \boldsymbol{\theta}, \sigma_\epsilon^2) - \lambda \|\boldsymbol{\beta}\|_1] \quad [6]$$

where $\lambda > 0$ is the tuning parameter. Estimation, shrinkage, and selection will be undertaken using a Gaussian link function in the R-package glmmLasso which combines gradient ascent optimisation with the Fisher scoring algorithm (Groll, 2011). Many of our predictors will be categorical and the standard LASSO approach would treat the levels of a given categorical

variable as individual variables. Therefore, for selection at the whole factor-level, we will use a modified version of the algorithm, i.e., the group Lasso, see Yuan and Lin (2006) for details.

1.2. Survival submodel

We define the observed 'failure' time for the ith patient as $T_i^* = \min(T_{1i}, T_{2i}, \ldots, T_{Gi}, C_i)$ where T_{gi} is the time to event type $g = 1,2,\ldots,G$ and C_i is the censoring time. We also define an event indicator δ_i, which equals 0 if censored, or g if $T_i^* = T_{gi}$. To model the competing secondary outcomes in the survival submodel, we use cause-specific hazards (Putter et al, 2007), where for patient i, the instantaneous rate of failure for event type g at time $t > 0$ is

$$\begin{aligned} h_{gi}(t|\mathcal{F}_i(t), \boldsymbol{w}_i(t)) &= \lim_{\Delta t \to 0} \Pr\{t \leq T_i^* < t + \Delta t, \delta_i = g \mid T_i^* \geq t, \mathcal{F}_i(t), \boldsymbol{w}_i(t)\} / \Delta t \\ &= h_{g0}(t) \exp\left[\boldsymbol{\gamma}_g{}' \boldsymbol{w}_i(t) + f\{\mathcal{F}_i(t), \mathbf{u}_i, \boldsymbol{\alpha}_g\}\right] \end{aligned} \quad [7]$$

where $\mathcal{F}_i(t) = \{y_i(s),\ 0 \leq s < t\}$ represents the patient's history of log serum creatinine measures up to time t, and $\boldsymbol{w}_i(t)$ is a vector of time-dependent covariates (that may or may not include components of $\mathbf{x}_i(t)$ and $\mathbf{z}_i(t)$. The function f specifies which components/features of the longitudinal submodel are included in the linear predictor of the survival submodel. We will compare candidate functional forms for f including:

- a linear function of the current log serum creatinine measurement: $\alpha y_i(t)$
- the rate of change (slope): $\alpha \frac{\mathrm{d}y_i(t)}{\mathrm{d}t}$
- a function of the accumulated process: $\alpha \int_0^t y_i(s)\mathrm{d}s$
- a function of the random effects: $\boldsymbol{\alpha}'\mathbf{u}_i$

The baseline hazard for event type g will be modelled flexibly using a B-splines approach as

$$\log h_{g0}(t) = \gamma_{h_{g0},0} + \sum_{q=1}^{Q_g} \gamma_{h_{g0},q} B_{gq}(t, \boldsymbol{v}_g) \quad [8]$$

where $B_{gq}(t, \boldsymbol{v}_g)$ is the qth basis function of a B-spline with knots $v_{g1}, \ldots, v_{gq}$ and $\boldsymbol{\gamma}_{h_{g0}}$ is a vector of coefficients. In the event that we are struggling with model convergence, a common baseline hazard function will be used across the different event types.

1.3. Estimation

To obtain estimates to be used as arguments in the initialisation of the joint optimisation process, the longitudinal and survival submodels are fit separately using standard approaches. We will apply a Bayesian approach to the estimation of the parameters in the joint modelling framework by optimising the posterior distribution of the model parameters and the random effects conditioning on the observed longitudinal and survival data. The posterior distribution is comparable to

$$p(\boldsymbol{\theta}, \mathbf{u}) \propto \prod_{i=1}^{M} \prod_{j=1}^{n_i} p(y_{ij} | \mathbf{u}_i; \boldsymbol{\theta}) p(T_i^*, \delta_i | \mathbf{u}_i; \boldsymbol{\theta}) p(\mathbf{u}_i | \boldsymbol{\theta}) p(\boldsymbol{\theta}) \quad [9]$$

where $\boldsymbol{\theta}$ is the combined longitudinal, survival, and random effects parameter vector, and $\mathbf{u}$ is the vector of random effects for the cohort. $p(y_{ij}|\mathbf{u}_i;\boldsymbol{\theta})$ is the density function for the longitudinal submodel, $p(\mathbf{u}_i|\boldsymbol{\theta})$ is the density function for the random effects, $p(\boldsymbol{\theta})$ is a prior distribution for the parameter vector, and $p(t,\delta_i|\mathbf{u}_i,\boldsymbol{\theta})$ is the density function for the competing risks survival process:

$$p(t,\delta_i|\mathbf{u}_i,\boldsymbol{\theta}) = \prod_{g=1}^{G}\left[h_{gi}(t|\mathcal{F}_i(t),\boldsymbol{w}_i(t);\boldsymbol{\theta})\{1-F_{gi}(t|\mathcal{F}_i(t),\boldsymbol{w}_i(t);\boldsymbol{\theta})\}\right]^{\mathrm{I}(\delta_i=g)} \times \left(1-\sum_{g=1}^{G}F_{gi}(t|\mathcal{F}_i(t),\boldsymbol{w}_i(t);\boldsymbol{\theta})\right)^{\mathrm{I}(\delta_i=0)} \quad [10]$$

where $F_{gi}(t|\mathcal{F}_i(t),\boldsymbol{w}_i(t);\boldsymbol{\theta}) = 1-\exp\left(-\int_0^t h_{gi}(s|\mathcal{F}_i(t),\boldsymbol{w}_i(t);\boldsymbol{\theta})\mathrm{d}s\right)$. Optimisation of [9], with respect to the vectors of parameters and random effects, will be undertaken using a Markov chain Monte Carlo algorithm. If the optimisation process proves to be too computationally demanding within the timeframe of the RENAL-HF project, we will try a two-step approach to the optimisation problem with an importance sampling correction, as described by Mauff and others (2020).

1.4. Extending to multiple longitudinal processes

Along with serum creatinine, other markers of interest include potassium and sodium levels. Direct longitudinal modelling of these markers on an individual basis (in the manner described in the previous sections) is not appropriate due to the excessive correlation between the markers that we would likely encounter. As such, we intend to use principal component analysis to generate new variables that are linear functions of the original variables, that successively maximise variance, and are uncorrelated with one another. The principal components can be obtained as the solution to an eigenvalue/vector problem or from the singular value decomposition of the centred (n×3) matrix $\mathbf{Y}$ where the columns $\mathbf{y}_1$, $\mathbf{y}_2$, and $\mathbf{y}_3$ correspond to the centred observations of serum creatinine, potassium, and sodium respectively. As such, for each principal component, we will have

$$\sum_{k=1}^{3} a_k\mathbf{y}_k = \mathbf{Ya}$$

where $\mathbf{a}$ is a vector of constants. The variance of the linear combination is

$$\mathrm{var}(\mathbf{Ya}) = \mathbf{a}'\mathbf{Sa}$$

where $\mathbf{S}$ is the covariance matrix for $\mathbf{Y}$.

Model fitting and prediction modelling will then be undertaken with the uncorrelated principal components treated as the longitudinal response variables. To preserve all the variance in the original vectors $\mathbf{y}_1$, $\mathbf{y}_2$, and $\mathbf{y}_3$, we will retain all three principal components and the joint regression model will be optimised with respect to the multivariate longitudinal principal components and the competing secondary endpoints. For the primary analyses, the predicted

pathways will then be back transformed for threshold analyses and scheduling activities specific to each $\mathbf{y}$.

2. Prediction

2.1. Predicting primary outcome

We are interested in predicting future values of log serum creatinine (i.e., the random variable $Y_{i,j+1}$) by conditioning on the outcome observation history $\mathbf{y}_{i1}, \mathbf{y}_{i2}, \dots, \mathbf{y}_{ij}$, the timing of the observations $t_{i1}, t_{i2}, \dots, t_{i,j+1}$ (assuming that $t_{i,j+1}$ is known), and the observed covariate history $\mathbf{x}_{i1}, \mathbf{x}_{i2}, \dots, \mathbf{x}_{ij}$ for that patient. We define $\boldsymbol{\mathcal{F}}_{ij}$ as the filtration of all observable information (serum creatinine and other covariates) up to and including the jth observation for the ith patient (i.e., time-point t_{ij}), we can specify the probability distribution for $Y_{i,j+1}|\boldsymbol{\mathcal{F}}_{ij}$ as

$$p_y\left(y_{i,j+1}|\boldsymbol{\mathcal{F}}_{ij}\right) = \int p_{y|u}(y_{i,j+1}|\boldsymbol{\mathcal{F}}_{ij}, \mathbf{u}_{ij}) p_u(\mathbf{u}_{ij}|\boldsymbol{\mathcal{F}}_{ij}) d\mathbf{u}_{ij} \qquad [11]$$

where, for simplicity we assume that $\mathbf{x}_{ij}$and $\mathbf{z}_{ij}$ provide adequate summaries of $\mathbf{x}_{i1}, \mathbf{x}_{i2}, \dots, \mathbf{x}_{ij}$ and $\mathbf{z}_{i1}, \mathbf{z}_{i2}, \dots, \mathbf{z}_{ij}$ respectively (i.e., we invoke a first-order Markov assumption), and accounting for the uncertainty in the estimation of $\boldsymbol{\beta}$, we have

$$Y_{i,j+1}|\boldsymbol{\mathcal{F}}_{ij}, \mathbf{u}_{ij} \sim N\left((\mathbf{F}_{i,j+1|j}\mathbf{x}_{ij})'\widehat{\boldsymbol{\beta}} + (\mathbf{B}_{i,j+1|j}\mathbf{z}_{ij})'\mathbf{u}_{ij},\ (\mathbf{F}_{i,j+1|j}\mathbf{x}_{ij})'\widehat{\boldsymbol{\Psi}}\mathbf{F}_{i,j+1|j}\mathbf{x}_{ij} + \sigma_\epsilon^2\right) \qquad [12]$$

where $\widehat{\boldsymbol{\Psi}}$ is the covariance matrix for the estimated vector of coefficients $\widehat{\boldsymbol{\beta}}$ and the matrices $\mathbf{F}$ and $\mathbf{B}$ are assumed to be deterministic updating functions. For example, given the elapsed time between observations, a simple updating of an intercept and time-based covariate vector, with $\mathbf{x}_{ij} = (1, t_{ij})'$, would be

$$\mathbf{x}_{i,j+1|j} = \mathbf{F}_{i,j+1|j}\mathbf{x}_{ij} = \begin{bmatrix} 1 & 0 \\ 0 & t_{i,j+1}/t_{ij} \end{bmatrix} \begin{bmatrix} 1 \\ t_{ij} \end{bmatrix} = \begin{bmatrix} 1 \\ t_{i,j+1} \end{bmatrix}$$

where $\mathbf{F}_{i,j+1|j} = \begin{bmatrix} 1 & 0 \\ 0 & t_{i,j+1}/t_{ij} \end{bmatrix}$.

A key issue is the estimation of the patient-specific random effects vector at time t_{ij} conditioning on the information observed up to that point, i.e., $\mathbf{u}_{ij}|\boldsymbol{\mathcal{F}}_{ij}$.

Proposed Option: Recursive estimation of $p_u(\mathbf{u}_{ij}|\boldsymbol{\mathcal{F}}_{ij})$

Rearranging equation [1], i.e., the jth log serum creatinine measurement for the ith patient, as

$$d_{ij} = \mathbf{z}'_{ij}\mathbf{u}_i + \epsilon_{ij} \qquad [13]$$

where $d_{ij} = y_{ij} - \mathbf{x}'_{ij}\widehat{\boldsymbol{\beta}}$ and defining the observation set $\mathbf{D}_{ij} = d_{i1}, \dots, d_{ij}$, we have

$$p_u\left(\mathbf{u}_{ij}|\boldsymbol{\mathcal{F}}_{ij}\right) = p_u\left(\mathbf{u}_{ij}|\mathbf{D}_{ij}\right) = \frac{p\left(d_{ij}|\mathbf{u}_{ij}\right)p\left(\mathbf{u}_{ij}|\mathbf{D}_{i,j-1}\right)}{p\left(d_{ij}|\mathbf{D}_{i,j-1}\right)} \qquad [14]$$

where

$$p\left(\mathbf{u}_{ij}|\mathbf{D}_{i,j-1}\right) = \int p\left(\mathbf{u}_{ij}|\mathbf{u}_{i,j-1}\right)p\left(\mathbf{u}_{i,j-1}|\mathbf{D}_{i,j-1}\right)d\mathbf{u}_{i,j-1} \qquad [15]$$

and the normalising denominator in [14] is

$$p(d_{ij}|\mathbf{D}_{i,j-1}) = \int p(d_{ij}|\mathbf{u}_{ij})p(\mathbf{u}_{ij}|\mathbf{D}_{i,j-1})d\mathbf{u}_{ij} \qquad [16]$$

Using the estimated covariance matrix $\widehat{\boldsymbol{\Sigma}}$, and initialising with $\widehat{\mathbf{u}}_{i,0|0} = \mathbf{0}$ and $\widehat{\boldsymbol{\Sigma}}_{i,0|0} = \widehat{\boldsymbol{\Sigma}}$, we can structure the solution to [11]-[16] as recursive prediction and updating stages using a Kalman filter. We can regard the random effects for a given patient as static latent variables that we attempt to quantify using the patient's measurement history. As such, our estimates will remain unchanged between serum creatinine measurement points and will then be updated as we incorporate a new measurement. We therefore have the prediction equations at the jth observation point for the ith patient as

$$\widehat{\mathbf{u}}_{i,j|j-1} = \widehat{\mathbf{u}}_{i,j-1|j-1} \qquad [17]$$

$$\widehat{\boldsymbol{\Sigma}}_{i,j|j-1} = \widehat{\boldsymbol{\Sigma}}_{i,j-1|j-1} \qquad [18]$$

Then, upon observing additional information y_{ij} and $\mathbf{x}_{ij}$, at the updating stage we have the innovation residual

$$\tilde{a}_{ij} = y_{ij} - \mathbf{x}'_{ij}\widehat{\boldsymbol{\beta}} - \mathbf{z}'_{ij}\widehat{\mathbf{u}}_{i,j|j-1} = d_{ij} - \mathbf{z}'_{ij}\widehat{\mathbf{u}}_{i,j|j-1} \qquad [19]$$

with variance

$$s_{ij} = \mathbf{z}'_{ij}\widehat{\boldsymbol{\Sigma}}_{i,j|j-1}\mathbf{z}_{ij} + \hat{\sigma}^2_{\epsilon}. \qquad [20]$$

The Kalman gain is therefore

$$\mathbf{K}_{ij} = [1/s_{ij}]\widehat{\boldsymbol{\Sigma}}_{i,j|j-1}\mathbf{z}_{ij}. \qquad [21]$$

The updated estimate of the random effects vector is

$$\widehat{\mathbf{u}}_{i,j|j} = \widehat{\mathbf{u}}_{i,j|j-1} + \mathbf{K}_{ij}\tilde{a}_{ij} \qquad [22]$$

and the updated estimate of the covariance matrix is

$$\widehat{\boldsymbol{\Sigma}}_{i,j|j} = (\mathbf{I} - \mathbf{K}_{ij}\mathbf{z}'_{ij})\widehat{\boldsymbol{\Sigma}}_{i,j|j-1} \qquad [23]$$

Therefore, we would have $\mathbf{u}_{ij}|\mathcal{F}_{ij} \sim N[\widehat{\mathbf{u}}_{i,j|j}, \widehat{\boldsymbol{\Sigma}}_{i,j|j}]$ in equation [11].

To extend by incorporating the uncertainty in the estimation of $\widehat{\boldsymbol{\beta}}$, the error term in [20] would be replaced by a linear combination of the observation (measurement) error and the uncertainty in the estimation of $\widehat{\boldsymbol{\beta}}$, i.e., $v_{ij} \sim N(0,\ \mathbf{x}'_{ij}\widehat{\boldsymbol{\Psi}}\mathbf{x}_{ij} + \sigma^2_{\epsilon})$ and the innovation variance would be

$$s_{ij} = \mathbf{z}'_{ij}\widehat{\boldsymbol{\Sigma}}_{i,j|j-1}\mathbf{z}_{ij} + \mathbf{x}'_{ij}\widehat{\boldsymbol{\Psi}}\mathbf{x}_{ij} + \hat{\sigma}^2_{\epsilon}.$$

Alternative option

Given the observed information up to the jth measurement point, we could estimate and then plug maximum likelihood estimates or empirical Bayes estimates of the random effects directly in equation (12). The empirical Bayes estimates are

$$\widehat{\mathbf{u}}_{ij} = \widehat{\boldsymbol{\Sigma}}\mathbf{Z}'_{ij}\widehat{\mathbf{V}}_{ij}^{-1}(\mathbf{y}_{ij} - \mathbf{X}_{ij}\widehat{\boldsymbol{\beta}}) \qquad [24]$$

where $\mathbf{y}_{ij} = (y_{i1}, \dots, y_{ij})'$, $\mathbf{X}_{ij} = (\mathbf{x}'_{i1}, \dots, \mathbf{x}'_{ij})'$, $\mathbf{Z}_{ij} = (\mathbf{z}'_{i1}, \dots, \mathbf{z}'_{ij})'$ and $\widetilde{\mathbf{V}}_{ij} = \mathbf{Z}_{ij}\widetilde{\boldsymbol{\Sigma}}\mathbf{Z}'_{ij} + \sigma_\epsilon^2\, \mathbf{I}_j$.
To extend this approach, we could specify a distribution for the empirical Bayes estimates using the techniques outlined in Bates and Pinheiro (1998).

2.2. Predicting secondary outcomes

Here we adapt the Monte Carlo process described in by Rizopoulos (2011) to our competing risks scenario. For a given patient i who has survived (and has longitudinal measurements) until time t_{ij}, the aim is to estimate the conditional probability of remaining event-free beyond a given horizon time Δ, e.g., one year. For event type g, the probability is

$$s_i\big(t_{ij} + \Delta \mid t_{ij}\big) = \Pr\big(T_{gi} > t_{ij} + \Delta \mid T_i^* > t_{ij}, \mathbf{x}_{ij}, \mathbf{z}_{ij}, \boldsymbol{\theta}\big) \qquad [25]$$

The survival probabilities can then be used to derive cause-specific risk scores for the event of interest within the interval $(t_{ij}, t_{ij} + \Delta)$.
For each repetition $r = 1, 2, \dots, R$, we draw $\widetilde{\boldsymbol{\beta}}^{(r)}$ from $\boldsymbol{\beta} \sim N(\widehat{\boldsymbol{\beta}}, \widehat{\boldsymbol{\Psi}})$, then condition on $\boldsymbol{\beta}^{(r)}$ to draw a realisation $\mathbf{u}_{ij}^{(r)}$ from the posterior distribution of the random effects, the derivation of which is described in the previous section. The probability of remaining free of event type k is then

$$s_i^{(r)}\left(t_{ij} + \Delta \mid \mathbf{u}_{ij}^{(r)}; \widetilde{\boldsymbol{\beta}}^{(r)}\right) = \frac{\left(\sum_{g=1}^{G} F_{gi}^{(r)}(\infty)\right) - F_{ki}^{(r)}(t_{ij} + \Delta) - \left(\sum_{g \neq k} F_{gi}^{(r)}(t)\right)}{\sum_{g=1}^{G}\left(F_{gi}^{(r)}(\infty) - F_{gi}^{(r)}(t)\right)} \qquad [26]$$

where, using the cumulative incidence function defined for equation [10], we have $F_{gi}^{(r)}(t) = F_{gi}\big(t \big| \boldsymbol{\mathcal{F}}_{ij}; \boldsymbol{\theta}^{(r)}\big)$.

3. Predictive accuracy

3.1. Primary outcome

Using the updated estimate of the random effects at the jth observation for the ith patient (equations [22] and [23]), we can produce one-step point predictions of log serum creatinine values and compare the exponents of the predictions to the observed serum creatinine measurements using

$$\begin{aligned}\text{RMSPE} &= \left((1/n)\sum_{i=1}^{M}\sum_{j=1}^{n_i}\left(e^{y_{i,j+1}} - e^{\hat{y}_{i,j+1|j}}\right)^2\right)^{1/2} \\ &= \left((1/n)\sum_{i=1}^{M}\sum_{j=1}^{n_i}\left(e^{y_{i,j+1}} - e^{(\mathbf{F}_{i,j+1|j}\mathbf{x}_{ij})'\widehat{\boldsymbol{\beta}} + (\mathbf{B}_{i,j+1|j}\mathbf{z}_{ij})'\widehat{\mathbf{u}}_{i,j|j}}\right)^2\right)^{1/2} \qquad [27]\end{aligned}$$

or, preferably, using the estimated distribution of the predicted response, as

$$\text{RMSPE} = \left((1/n) \sum_{i=1}^{M} \sum_{j=1}^{n_i} \int \left(e^{y_{i,j+1}} - w_{i,j+1|j} \right)^2 p\left(w_{i,j+1|j} \middle| \boldsymbol{\mathcal{F}}_{ij} \right) dw_{i,j+1} \right)^{1/2} \quad [28]$$

If we plug empirical Bayes estimates of the random effects directly in equation [28], we have the conditional distribution for $W_{i,j+1|j} = \exp(Y_{i,j+1|j})$ as

$$W_{i,j+1} | \boldsymbol{\mathcal{F}}_{ij}, \widehat{\mathbf{u}}_{ij} \sim \text{Lognormal}\left((\mathbf{F}_{i,j+1|j} \mathbf{x}_{ij})' \widehat{\boldsymbol{\beta}} + (\mathbf{B}_{i,j+1|j} \mathbf{z}_{ij})' \widehat{\mathbf{u}}_{ij},\ (\mathbf{F}_{i,j+1|j} \mathbf{x}_{ij})' \widehat{\boldsymbol{\Psi}} \mathbf{F}_{i,j+1|j} \mathbf{x}_{ij} + \sigma_\epsilon^2 \right)$$

However, if we rewrite [28] as

$$\text{RMSPE} = \left((1/n) \sum_{i=1}^{M} \sum_{j=1}^{n_i} \int \left(e^{y_{i,j+1}} - e^{\psi} \right)^2 p_y\left(\psi \middle| \boldsymbol{\mathcal{F}}_{ij} \right) d\psi \right)^{1/2} \quad [29]$$

where p_y is given by [11], and we incorporate the posterior random effects distribution $\mathbf{u}_{ij} | \boldsymbol{\mathcal{F}}_{ij} \sim N(\widehat{\mathbf{u}}_{i,j|j}, \widehat{\boldsymbol{\Sigma}}_{i,j|j})$, with $\widehat{\mathbf{u}}_{i,j|j}$ and $\widehat{\boldsymbol{\Sigma}}_{i,j|j}$ given by [22] and [23] respectively, the solution to [29] will require a numerical approach, e.g., adaptive quadrature.

3.2. Secondary outcomes

When assessing predictive accuracy for the secondary outcomes, we need to incorporate the impact of censoring via loss to follow-up. The inverse probability of weighting approach uses Kaplan-Meier or Cox models for the censoring distribution. However, the censoring mechanism may be dependent on the longitudinal process, meaning that the censoring weights may be biased. Therefore, we will calculate predictive accuracy measures using joint model-based weights that account for the censoring distribution and can incorporate a dependency on the longitudinal process.

Discrimination

Using the probability of remaining free of event type g over the interval $(t, t + \Delta]$, as defined in equation [25], and randomly selecting a pair of patients $\{i, k\}$, where both remained event free at time t, but one goes on to experience event type g in $(t, t + \Delta]$ and the other does not, we would expect the model to assign a higher probability of remaining free of event type g to the patient that did not experience the event. As such, the discriminative ability of the joint model can be assessed using the area under the receiver operating characteristic curve (AUC) as

$$\text{AUC}(t, \Delta) = \Pr\left[s_i(t + \Delta \mid t) < s_j(t + \Delta \mid t) \mid \{T_i^* \in (t, t + \Delta]\} \cap \{T_k^* > t + \Delta\} \right] \quad [30]$$

In essence, the AUC involves counting the pairs of concordant patients. However, the presence of both censoring and competing risks means that not all pairs of patients can be ordered with respect to their time-to-event type g.

To account for competing event types, we will determine event type-specific AUC measures where patients experiencing the event of interest in $(t, t + \Delta]$ are compared against both those experiencing a competing event in $(t, t + \Delta]$ and those that remain free of all event types at $t +$

Δ. The AUC will be decomposed into component parts where the first part refers to the pairs of subjects who are comparable (with event times that can be ordered) with indicator function

$$\Omega_{ik}^{(1)}(t) = \quad [\{T_i^* \in (t, t+\Delta]\} \cap \{\delta_i = g\}] \\ \cap\, [(T_k^* > t+\Delta) \cup (\{T_k^* \in (t, t+\Delta]\} \cap \{\delta_i \neq g\})] \qquad [31]$$

For the set of comparable patients i and k, we can directly compare their survival probabilities and the component estimator is the proportion of concordant patients out of the set of comparable patients. The other parts of the decomposition consist of sets of patient combinations where, due to censoring or the occurrence of competing events, the patients cannot be directly compared. Patients in these sets contribute to the AUC via the incorporation of probability weights to induce comparability.

Calibration

The accuracy of predictions will be assessed using calibration plots, the calibration slope, and the integrated calibration index using techniques described by Austin and others (2020). To summarise predictive accuracy over proposed intervals of 1, 2, and 5 years from baseline (i.e., from heart failure diagnosis), we will use an approximation of the integrated Brier score. The Brier score evaluates predictive accuracy at a specific landmark time t and prediction horizon time t +Δ as

$$BS(t, \Delta) = \mathbb{E}[(D(t,\Delta) - s(t+\Delta|t))^2 \,|\, T > t] \qquad [32]$$

where $D_i(t, \Delta) = 1$ if the patient experiences the event of interest between time t and $t + \Delta$, and 0 if the patient experiences a competing event between t and $t + \Delta$ or remains event-free at time $t + \Delta$. The Brier score estimator is

$$\widehat{BS}(t, \Delta) = \frac{1}{M_t} \sum_{i=1}^{M_t} (D_i(t,\Delta) - \hat{s}_i(t+\Delta|t))^2 \qquad [33]$$

where M_t is the number of patients remaining in the risk set at time t. However, to summarise predictive accuracy over an interval $(t, t + \Delta)$, we will use the integrated version of the score where the integral will be approximated using Simpson's rule.

To account for misspecification of the fitted joint model, we will calculate internal-externally cross-validated versions of the measures of predictive accuracy discussed in this section.

4. Scheduling

4.1. Definition of WRF

Worsening renal function is defined as

- a 25% increase in serum creatinine within 12 months, or
- a 26.5μmol/L increase in serum creatinine within 12 months

A complicating factor is that the serum creatinine measurement at the current time point (i.e., t_j) may be an intermediate measurement on an already increasing trajectory. As such, we also need to consider the magnitude of the increase from previous measurement points if they occurred within the 12 months prior to the current measurement point.

4.2. Defining thresholds

At the jth serum creatinine measurement point (for the ith patient), we define j potential threshold values (for $k = 1, \dots, j$) as

$$c_{ik} = \begin{cases} \ln(\min\{ae^{y_{ik}}, e^{y_{ik}} + b\}) & \text{if } t_{ij} - t_{ik} < \Delta \\ \infty & \text{otherwise} \end{cases} \quad [34]$$

where $a = 1.25$, $b = 26.5$, and $\Delta = 1$ if the timescale is measured in years.

4.3. Estimating the probability of WRF

The objective is to estimate the probability of WRF within a given period from the current observation point (i.e., $t_{ij} + \Delta$) using Monte Carlo simulation. For each repetition $r = 1, 2, \dots, R$, we simulate $\tilde{\boldsymbol{\beta}}^{(r)}$ from $\boldsymbol{\beta} \sim N(\hat{\boldsymbol{\beta}}, \hat{\boldsymbol{\Psi}})$, then condition on $\boldsymbol{\beta}^{(r)}$ to simulate $\mathbf{u}_{ij}^{(r)}$ from the posterior distribution of the random effects. For $t > t_{ij}$ and $k = 1, \dots, j$, we define

$$\phi_{ik}^{(r)} = \inf_{t}.\left\{\tilde{g}_{ij}^{(r)}(t) \geq c_{ik}\right\} \quad [35]$$

where $\tilde{g}_{ij}^{(r)}(t) = \mathrm{E}\left[y_i(t) | \boldsymbol{\mathcal{F}}_{ij}; \boldsymbol{\beta}^{(r)}, \mathbf{u}_{ij}^{(r)}\right]$ is the expected pathway of log serum creatinine from the jth measurement point onwards. The minimum stopping time (or first threshold 'hitting time') from t_{ij} is then

$$\tau^{(r)} = \min\left\{\phi_{ik}^{(r)} / \mathrm{I}\left[\phi_{ik}^{(r)} - t_k \leq \Delta\right]\right\} \quad [36]$$

The empirical distribution function for the time-to-WRF is defined as

$$\hat{F}_{ij}(t) = (1/R)\sum_{r=1}^{R} \mathrm{I}\left[\tau^{(r)} \leq t\right] \quad [37]$$

We can also use the distribution of the expected values at $t_{i,j+1}$, i.e., $\tilde{g}_{ij}^{(r)}(t_{i,j+1})$, for $r = 1, 2, \dots, R$, to construct an empirical distribution for $p(w_{i,j+1|j} | \boldsymbol{\mathcal{F}}_{ij})$ in equation [28].

4.4. Utility function

Figure 1 illustrates the scenario for a specific patient with a longitudinal series of serum creatinine measurements up to their current renal monitoring point at time t_j. We are interested in WRF events within the interval $(t_j, t_j + \Delta]$. If the probability of remaining event free is below a specific threshold $c(t_j)$, action should be taken (e.g., stopping a medication or titrating the dose). If the probability of remaining event free is greater than the threshold, then we seek to schedule the next renal monitoring visit via a trade-off between the extra

information an additional measure of serum creatinine would provide and the potential cost of delaying the visit (i.e., the increased probability that the patient will experience WRF).

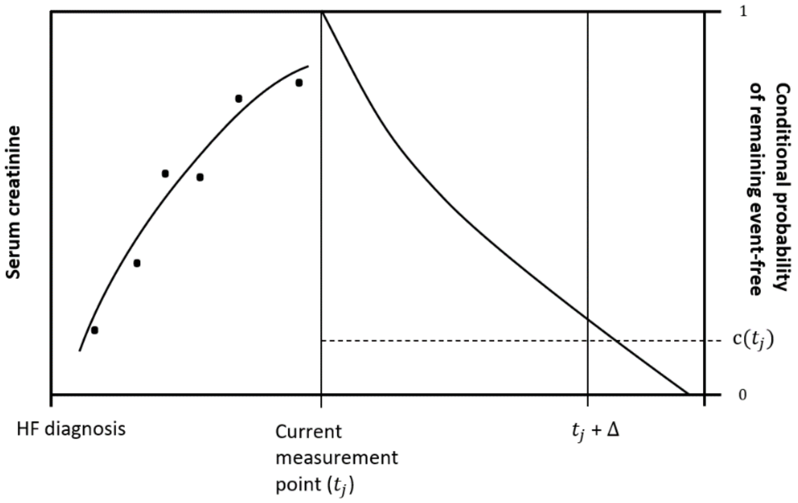

Figure 1. Illustrating a specific patient's longitudinal serum creatinine trajectory and their conditional probability of remaining free from WRF [Figure adapted from Rizopoulos et al, 2016].

Defining T_i as the time at which WRF occurs for the ith patient, and assuming that the patient has survived and contributed longitudinal measures up to their jth renal monitoring point (at time t_{ij}), scheduling of their subsequent visit will involve maximising the utility function (adapted from Rizopoulos et al, 2016):

$$\mathrm{U}(u|t_{ij}) = \mathbb{E}\left\{\lambda_1 \log\left(\frac{p(T_i|T_i > u, \{\mathcal{F}_i(t_{ij}), y_i(u)\}, \tilde{\mathbf{x}}_i(u), \tilde{\mathbf{z}}_i(u), \widehat{\boldsymbol{\theta}})}{p(T_i|T_i > u, \mathcal{F}_i(t_{ij}), \mathbf{x}_{ij}, \mathbf{z}_{ij}, \widehat{\boldsymbol{\theta}})}\right) - \lambda_2 \mathrm{I}[T_i > u]\right\} \quad [38]$$

where $\mathcal{F}_i(t_{ij}) = \{y_i(t_{ij});\ 0 \leq t_{ik} \leq t_{ij}, k \leq j, j = 1, \ldots, n_i\}$ represents their history of log serum creatinine measures, and, as previously specified, $\mathbf{x}_{ij}$ and $\mathbf{z}_{ij}$ are the design vectors for the fixed and random components of the longitudinal submodel (consisting of functions of time, baseline, and time-dependent covariates), $\widehat{\boldsymbol{\theta}}$ is the set of estimated parameters for the joint model, the vectors $\tilde{\mathbf{x}}_i(u)$ and $\tilde{\mathbf{z}}_i(u)$ are deterministically time-updated functions of $\mathbf{x}_{ij}$ and $\mathbf{z}_{ij}$ (as proposed in §2.1), and λ_1 and λ_2 are constants that facilitate the trade-off between the two components of [38] and are to be estimated from the data or specified following discussions with our clinical advisors.

The first term in [38] represents the gain in information about T_i we would expect if we scheduled an additional visit for renal monitoring at time $u > t_{ij}$. The information gain at u would be 0 if the true WRF time occurred before u, i.e., $T_i < u$, which would be determined directly by the fact that $y_i(u)$ had breached the prospective WRF threshold defined at t_{ij}. If the WRF threshold had not been breached, the new measurement $y_i(u)$ would provide more information on when, and if, a breach might occur. It follows that the expected gain in

information would be greater the further u is from t_{ij}. However, as we are forecasting from the current monitoring point at time t_{ij}, we are simply deriving projections of $y_i(u)$. By aggregating over a large number of said projections, we are able to estimate the expected gain in information that would be acquired by monitoring at time u.

Taking the expectation of the second term in equation [38] produces the conditional probability that the patient does not experience WRF before time u as

$$\mathbb{E}\{\mathrm{I}[T_i > u]\} = \Pr\big(T_i > u | T_i > t_{ij} \mathcal{F}_i(t_{ij}), \widehat{\boldsymbol{\theta}}\big) = 1 - \hat{F}_{ij}(t) \quad [39]$$

where $\hat{F}_{ij}(t)$ is the empirical cumulative incidence for WRF described in equation [37]. As such, the second term in [38] can be regarded as the 'cost' of waiting.

If we assume that, at time t_{ij}, the patient has already been allocated to a monitoring scheme and the subsequent monitoring visit is planned at time $t_{i,j+1}$, we aim to either adhere to or override this scheme. The optimal monitoring time will then be found using a Monte Carlo simulation scheme to maximise the first term in [38] over the interval $(t, \varphi]$ where

$$\varphi = \min\,(u\text{: } 1 - \hat{F}_{ij}(t) = c(t_{ij}), t_{ij})$$

4.5. Incorporating the secondary outcomes

In the previous sub-section, we assume that the scheduling decision is based solely on the desire to avoid WRF. However, if we wish to incorporate the secondary competing outcomes in the scheduling decision, we need to consider either: (a) constructing and optimising utility functions for WRF and each of the secondary outcomes separately, then selecting the optimal time for monitoring u as the minimum of the recommendations from the individual models; (b) modifying [38] so that, in the first term, the event time $T_i^* = \min\,(T_{0i}, T_{1i}, T_{2i}, \dots, T_{Gi})$ is the first to occur in the set of $G + 1$ competing event types (where T_{0i} is the event time for WRF). Under option (b), the functional form of the first term would remain the same but the second term will be replaced by the corresponding event-specific probabilities (see equation [25]).

5. Alternative approaches

1. By re-formulating equation (1) and fitting the mixed effects model as a one-step prediction equation, we can bypass several issues that arise when attempting to make predictions with the fitted model. At time t_{ij}, the jth log serum creatinine measurement for the ith patient is represented as an updated function of the information ($\mathbf{x}_{i,j-1}$ and $\mathbf{z}_{i,j-1}$) that was available at the previous measurement point, as

$$y_{ij} = (\mathbf{F}_{i,j|j-1}\mathbf{x}_{i,j-1})'\widehat{\boldsymbol{\beta}} + (\mathbf{B}_{i,j|j-1}\mathbf{z}_{i,j-1})'\mathbf{u}_i + \epsilon_{ij} \quad [40]$$

where $\mathbf{F}$ and $\mathbf{B}$ are the deterministic updating functions introduced in equation [12], i.e., equation [40] is a lagged version of [1]. This arrangement potentially facilitates: (i) improved

parameterisation of $\boldsymbol{\beta}$ with regard to the primary goal of applying the model in a predictive capacity; (ii) a simpler variance structure for the prediction equations.

2. An alternative approach would be to identify instances of WRF in the training data. Interpolation could then be used to estimate the point (between successive serum creatinine measurement points) at which the threshold was breached. Time-to-event models could then be fitted to the data. This would provide a more direct route to the estimation of the probability of WRF within a given period.

3. An alternative approach to the scheduling problem discussed in §4, with respect to the primary outcome, would be to simply apply a cut-off to the empirical distribution for the probability of remaining event free, i.e., the optimal scheduling time would be the point at which the probability reached the lower bound of acceptability, if this optimal time is specified to occur before the next pre-planned visit.

References

Austin PC, Harrell FE, van Klaveren D (2020) Graphical calibration curves and the integrated calibration index (ICI) for survival models. Statist. Med., 39: 2714–42.

Bates DM, Pinheiro JC (1998) Computational methods for multilevel modelling. In Technical Memorandum BL0112140-980226-01TM. Murray Hill, NJ: Bell Labs, Lucent Technologies.

Groll A (2011) glmmLasso: Variable Selection for Generalized Linear Mixed Models by L1-penalized Estimation. R package version 1.0.1

Mauff K, Steyerberg E, Kardys I, Boersma E, Rizopoulos D (2020) Joint models with multiple longitudinal outcomes and a time-to-event outcome: a corrected two-stage approach. Stat. Comput., 30: 999–1014.

Putter H, Fiocco M, Geskus RB (2007) Tutorial in biostatistics: competing risks and multi-state models. Statist. Med., 26: 2389-430.

Rizopoulos D (2011) Dynamic predictions and prospective accuracy in joint models for longitudinal and time-to-event data. Biometrics, 67: 819-29.

Rizopoulos D, Taylor JMG, van Rosmalen J, Steyerberg EW, Takkenberg JJM (2016) Personalized screening intervals for biomarkers using joint models for longitudinal and survival data. Biostatistics, 17: 149-64.

Tibshirani R (1996) Regression shrinkage and selection via the lasso. J. R. Stat. Soc. B, 58: 267–288.

Yuan M, Lin Y (2006) Model selection and estimation in regression with grouped variables. J. R. Stat. Soc. B, 68: 49–67.

Net Benefit

Threshold range: In the context of renal monitoring, the decision to intervene (e.g. to call in a patient for an early blood test or to stop treatment) is likely to be made with a lower risk probability than in the case of high-risk surgery. A clinically relevant range of 10-30 percent likelihood of developing WRF is pre-specified. This reflects the clinical reality that a risk of 10 percent for significant renal decline justifies a low-cost blood test, while a risk of more than 30 percent may require a change in the medicine.

Net Benefit (NB) is calculated as:

$$\frac{TP}{N} - \frac{FP}{N} \times \frac{p_t}{1 - p_t}$$

where p_t is the threshold probability. These metrics balance the benefits of the discovery of a real case (TP) against the damages of false-positive interventions (FP). For this study, we assess the net benefit across a pre-defined threshold probability range of 0.10 to 0.30, which represents the clinical window within which primary care physicians would consider changing the frequency of monitoring or the dose of the medication.
