# Supplementary material for: Development and validation of clinical prediction models for personalized renal function monitoring in people with heart failure in primary care: the RENAL-HF study protocol
Source: Eur Heart J Digit Health. 2026 Mar 31;7(4):ztag055. doi: 10.1093/ehjdh/ztag055 (PMC13131987; doi:10.1093/ehjdh/ztag055)
Supplement: ztag055_Supplementary_Data [file ztag055_supplementary_data.zip › S4_updated.pdf]

Supplementary File 4 : RNN-LSTM model

A multi-layered LSTM model will be created to forecast deteriorating renal function in heart failure patients. A dense output layer will come after two hidden LSTM layers, each of which has 64 hidden units, in the neural network architecture. Demographics, comorbidities, longitudinal biochemical parameters, and medication records will all be used as input data for model building. To improve computational efficiency and model performance, we will implement feature selection using two complementary techniques: Randomized Dependence Coefficient (RDC) analysis and Random Forest Recursive Feature Elimination (RF-RFE). The RDC method will quantify non-linear dependencies between variables and the outcome, while RF-RFE will iteratively remove the least important features based on random forest importance metrics. Each patient record will be represented as a matrix of features across time points, and patients with at least three consecutive creatinine measurements will be used to train the model. The Adam optimizer will be used to optimize the model, with mean squared error serving as the loss function and an initial learning rate of 0.001. To avoid overfitting, we will employ early stopping based on validation loss with a 10-epoch patience and dropout regularization (rate=0.2) between LSTM layers. The LSTM model will be developed using Keras architecture in R. Hyperparameters and the final model architecture will be established by cross-validation on the development dataset.

Variable Transformation Strategy: Transformation is applied conditionally according to the variable distribution. Skewed biomedical distributions (e.g. serum creatinine, urea) are transformed in a logarithmic manner to reduce outliers and to facilitate gradient descent. Variables that are normally distributed (e.g., sodium) are standardised (standardisation of the Z-score).

DYNAMIC RENAL TEST RECOMMENDATION SYSTEM (DRTRS) – Proof-of-concept

```
# Load necessary libraries and data
library(dplyr)

#Data resource
data <- EHR_MASTER_DATA # Database

# Define function for calculating eGFR
calculate_egfr <- function(creatinine, age, gender, ethnicity) {
  # Code for calculating eGFR using the Renal-HF equation
  calculation
} #

#Set today date
Current_date = 'Today' #current sys.time()

#Define function for recommending the time to the next blood test
recommend_next_test <- function(patient_id, data) {
  patient_data <- data %>%
    filter(patient_id == patient_id)

  #t+1 predicted by main model (RNN-LSTM)******
  latest_creatinine <-RNN_LSTM_SCr

  #predicting eGFR based on creatinine value predicted by LSTM
  Predicted_egfr <- calculate_egfr(latest_creatinine,
                                patient_data$age[length(patient_data$age)],
                                patient_data$gender[length(patient_data$gender)],

patient_data$ethnicity[length(patient_data$ethnicity)])

  if (Predicted_egfr >= 60) {
    # Patient has normal kidney function
    # Recommend next blood test in 6-12 months
    next_test_time <- as.Date(Sys.Date() + sample(182:365, 1), origin =
Current_date)
    return(next_test_time)
  }
      else if (Predicted_egfr >= 45 && Predicted_egfr < 60) {
    # Patient has mild to moderate kidney disease
    # Recommend next blood test in 6 months
    next_test_time <- as.Date(Sys.Date() + 182, origin = Current_date)
    return(next_test_time)
  }
      else if (Predicted_egfr >= 30 && Predicted_egfr < 45) {
    # Patient has moderate to severe kidney disease
    # Recommend next blood test in 3 months
    next_test_time <- as.Date(Sys.Date() + 91, origin = Current_date)
    return(next_test_time)
  }
      else if (Predicted_egfr >= 15 && Predicted_egfr < 30) {
    # Patient has severe kidney disease
    # Recommend next blood test in 1 month
    next_test_time <- as.Date(Sys.Date() + 7, origin = Current_date)
    return(next_test_time)
  }
      else if (Predicted_egfr < 15) {
    # Patient has kidney failure
    # Recommend next blood test immediately
    next_test_time <- as.Date(Sys.Date() + 0, origin = Current_date)
    return(next_test_time)
  }
      else {
```

```
    # should never occur
    return('error found')
  }
}

# Set up a loop to recommend the time to the next blood test for each patient
while (TRUE) {
  # Get the latest data for each patient
  latest_data <- data %>%
    group_by(patient_id) %>%
    slice_tail(n = 5) %>%  # takes latest 5 records.need to modify for patient less
records.
    ungroup()
  # Recommend the time to the next blood test for each patient
  patient_ids <- unique(latest_data$patient_id)
  for (patient_id in patient_ids) {
    next_test_time <- recommend_next_test(patient_id, latest_data)
    print(paste("Next blood test for patient", patient_id, "should be
done on", next_test_time))
  } #
 # Wait 24 hours before recommending the next blood test again
  Sys.sleep(86400) # could be changed weekly/monthly etc..,
}
#==================================END============================================
```
