# Supplementary material for: Development and validation of clinical prediction models for personalized renal function monitoring in people with heart failure in primary care: the RENAL-HF study protocol
Source: Eur Heart J Digit Health. 2026 Mar 31;7(4):ztag055. doi: 10.1093/ehjdh/ztag055 (PMC13131987; doi:10.1093/ehjdh/ztag055)
Supplement: ztag055_Supplementary_Data [file ztag055_supplementary_data.zip › S5.pdf]

Supp 5. RENAL-HF- Procedure used to identify potential assessment criteria

To identify potential assessment criteria for choosing between the models, we took the following steps.

First, we consulted the TRIPOD+AI statement (updated guidance for reporting clinical prediction models that use regression or machine learning methods) [1], the DECIDE-AI statement (Reporting guideline for the early stage clinical evaluation of decision support systems driven by artificial intelligence) [2], and the guidelines for developing and reporting machine learning predictive models in biomedical research developed by Luo et al. [3]. From each of these, we extracted items related to characteristics that could reasonably be expected to differ between the models. For instance, we ignored items related to the development dataset, as both RENAL-HF models used the same development dataset. The three lists of extracted items are provided in Table 1.

**TRIPOD+AI**	
Predictors	Clearly define all predictors, including how and when they were measured
Fairness	Describe any approaches that were used to address model fairness and their rationale
Model performance	Report model performance estimates with confidence intervals, including for any key subgroups (eg, sociodemographic).
Model updating	Report the results from any model updating, including the updated model and subsequent performance
Usability of the model in the context of current care	Describe how poor quality or unavailable input data (eg, predictor values) should be assessed and handled when implementing the prediction model<br>Specify whether users will be required to interact in the handling of the input data or use of the model, and what level of expertise is required of users<br>Discuss any next steps for future research, with a specific view to applicability and generalisability of the model
**DECIDE-AI**	
Implementation	a) Describe the settings in which the AI system was evaluated<br>b) Describe the clinical workflow/care pathway in which the AI system was evaluated, the timing of its use, and how the final supported decision was reached and by whom
Safety and errors	Provide a description of how significant errors/malfunctions were defined and identified
Human Factors	Describe the human factors tools, methods or frameworks used, the use cases considered, and the users involved
Ethics	Describe whether specific methodologies were utilised to fulfil an ethics-related goal (such as algorithmic fairness)<br>and their rationale

Subgroups analysis	Report on the differences in the main outcomes according to the prespecified subgroups
**Guidelines Luo et al.**	
Build the predictive model	Balance between model accuracy and model simplicity or interpretability<br>Familiarity with the modeling techniques of the end user<br>Potential pitfalls in interpreting the model

Table 1. Extracted items from TRIPOD+AI [1], DECIDE-AI [2], and the Luo et al. guidelines [3].

In the second step we eliminated overlap between the three lists and reformulated the items to make them applicable in a model selection context. For instance, we integrated the "Predictors" item from TRIPOD+AI with the "Implementation" item from DECIDE-AI. We also renamed some of the items to emphasize their role in the model selection process. For instance, "Model updating" from TRIPOD+AI became "Sustainability". The resulting list is shown in Table 2.

**Criterion**	**Description**
Model performance	How well does the model perform, including for any key subgroups (eg, sociodemographic)?
Fairness	Does the model provide equitable predictions across different groups (defined by characteristics such as gender, ethnicity, or socioeconomic status)?
Implementability	Are predictors readily accessible in routine primary care without requiring additional specialist tests?<br>How and when should they be measured?<br>How much computational resources will the model need?<br>Can the model be readily incorporated into decision-making processes?
Sustainability	What is the effort and cost involved for maintaining the model?
Interpretability	Is the model interpretable, and therefore easy to debug and to screen for biases?
Parsimony	What is the complexity of the model?
Generalisability	Will the model perform well on other datasets and in different contexts (e.g., different clinical systems)?

Table 2. Amalgamated and rephrased list of criteria from Table 1.

Finally, based on discussions in the consortium we decided to add one further potential model selection criterion, called *Novelty/Innovation*. This criterion evaluates the originality and innovativeness of the prediction models. It considers whether a model introduces new techniques, methodologies, or approaches that represent advancements over existing methods.

The definition of the criteria used for model selection is given below.

Accuracy: This criterion assesses how well the prediction models perform in terms of making accurate predictions of future serum creatinine levels and is measured using the root mean squared prediction error (RMSPE) on the data used to train the models and on independent data. A more accurate model will have smaller errors, indicating that its predictions are closer to the actual values.

Fairness: This criterion refers to the principle that the model should provide equitable predictions across different groups, often defined by characteristics such as gender, ethnicity, or socioeconomic status. Assessing fairness involves examining whether the model's predictions are equally accurate in each group.

Generalisability: This criterion refers to the extent to which the accuracy of a prediction model is retained beyond the context in which it was developed. A model with high generalisability performs well not only on the dataset it was trained on but also on other datasets or in different contexts (e.g., different clinical systems).

Implementability: This criterion assesses how easily a prediction model can be put into practice. Models that are easy to understand, require fewer computational resources, and can be readily incorporated into decision-making processes are deemed more implementable.

Interpretability: In the context of RENAL-HF, we do not aim to present case-specific explanations to end-users. The criterion here refers to the interpretability of the models in their entirety. A model that is more interpretable will be easier to debug (if something goes wrong or the model starts making incorrect predictions) and easier to screen for biases (e.g., towards or against any group of patients).

Novelty/Innovation: This criterion evaluates the originality and innovativeness of the prediction models. It considers whether a model introduces new techniques, methodologies, or approaches that represent advancements over existing methods.

Parsimony: This criterion refers to the principle of preferring simpler models over more complex ones when both achieve similar levels of performance. This is because simpler models are easier to interpret, generalise better to unseen data, and are less prone to overfitting (where the model fits the training data too closely but performs poorly on new data). Parsimony can be assessed in terms of the number of input variables, number of model terms, and number of estimated parameters.

Sustainability: When we choose a model, we need to make sure that it will continue to be effective and accurate as things change. This includes how simple it is to update the model when new information becomes available, and how much effort and cost are involved in maintaining it. For a model to be sustainable, it should adapt smoothly to changes without needing constant overhauls or excessive resources.

References

[1] Collins GS, Moons KGM, Dhiman P, Riley RD, Beam AL, Van Calster B, et al. TRIPOD+AI statement: updated guidance for reporting clinical prediction models that use regression or machine learning methods. BMJ. 2024 Apr 16;385:e078378. doi: 10.1136/bmj-2023-078378.

[2] Vasey B, Nagendran M, Campbell B, Clifton DA, Collins GS, Denaxas S, et al. Reporting guideline for the early-stage clinical evaluation of decision support systems driven by artificial intelligence: DECIDE-AI. Nat Med. 2022 May;28(5):924-933. doi: 10.1038/s41591-022-01772-9.

[3] Luo W, Phung D, Tran T, Gupta S, Rana S, Karmakar C, et al. Guidelines for developing and reporting machine learning predictive models in biomedical research: A multidisciplinary view. J Med Internet Res. 2016 Dec 16;18(12):e323. doi: 10.2196/jmir.5870.
